# A Comparison of Machine Learning Algorithms and Feature Sets for Automatic Vocal Emotion Recognition in Speech

**DOI:** 10.3390/s22197561

**Published:** 2022-10-06

**Authors:** Cem Doğdu, Thomas Kessler, Dana Schneider, Maha Shadaydeh, Stefan R. Schweinberger

**Affiliations:** 1 Department of Social Psychology, Institute of Psychology, Friedrich Schiller University Jena, Humboldtstraße 26, 07743 Jena, Germany; 2 Michael Stifel Center Jena for Data-Driven and Simulation Science, Friedrich Schiller University Jena, 07743 Jena, Germany; 3 Social Potential in Autism Research Unit, Friedrich Schiller University Jena, 07743 Jena, Germany; 4 DFG Scientific Network “Understanding Others”, 10117 Berlin, Germany; 5 Computer Vision Group, Department of Mathematics and Computer Science, Friedrich Schiller University Jena, 07743 Jena, Germany; 6 Department of General Psychology and Cognitive Neuroscience, Friedrich Schiller University Jena, Am Steiger 3/Haus 1, 07743 Jena, Germany; 7 German Center for Mental Health (DZPG), Site Jena-Magdeburg-Halle, 07743 Jena, Germany

**Keywords:** machine learning, vocal emotion recognition, speech, emotional speech database, feature set

## Abstract

Vocal emotion recognition (VER) in natural speech, often referred to as speech emotion recognition (SER), remains challenging for both humans and computers. Applied fields including clinical diagnosis and intervention, social interaction research or Human Computer Interaction (HCI) increasingly benefit from efficient VER algorithms. Several feature sets were used with machine-learning (ML) algorithms for discrete emotion classification. However, there is no consensus for which low-level-descriptors and classifiers are optimal. Therefore, we aimed to compare the performance of machine-learning algorithms with several different feature sets. Concretely, seven ML algorithms were compared on the Berlin Database of Emotional Speech: Multilayer Perceptron Neural Network (MLP), J48 Decision Tree (DT), Support Vector Machine with Sequential Minimal Optimization (SMO), Random Forest (RF), k-Nearest Neighbor (KNN), Simple Logistic Regression (LOG) and Multinomial Logistic Regression (MLR) with 10-fold cross validation using four openSMILE feature sets (i.e., IS-09, emobase, GeMAPS and eGeMAPS). Results indicated that SMO, MLP and LOG show better performance (reaching to 87.85%, 84.00% and 83.74% accuracies, respectively) compared to RF, DT, MLR and KNN (with minimum 73.46%, 53.08%, 70.65% and 58.69% accuracies, respectively). Overall, the emobase feature set performed best. We discuss the implications of these findings for applications in diagnosis, intervention or HCI.

## 1. Introduction

Vocal emotion recognition (VER) is a crucial part of the socio-emotional skill set that allows humans to understand others’ affective states within multimodal emotion processing. Together with visual signals and the verbal content of speech, nonverbal signals are also crucial for us to understand the emotional situation in a social context and therefore to adjust our behaviors to react appropriately. For the past two decades, areas within computer science such as Human Computer Interaction (HCI) have aimed to provide tools for automatic emotion recognition in natural speech [1]. Automatic emotion recognition tools are increasingly important in a growing range of applications. For instance, these methods can offer benefits to clinical diagnosis and intervention, such as in the fields of autism [2,3], prognosis and prevention (e.g., in the detection of depression or suicidal tendencies [4,5], or even in the context of therapies and human conflict resolution [6,7,8]. Such methods may also support better understanding of temporal causality of nonverbal behavior in dyadic human social interactions [9] or help to provide a non-invasive yet objective assessment of imitation behavior [10,11]. In the context of robotics and HCI, and especially when combined with high-quality speech synthesis technology [12], efficient emotion recognition algorithms can also contribute to experiencing interactions with robot or other technological devices as rewarding, seamless and trustworthy. At the same time, there is a relative gap in systematic research regarding the relative efficiency of these methods to classify specific emotions. Accordingly, the main aim of the present paper—a comparison of such algorithms—seems to be relevant for a range of applications.

Several classical machine-learning (ML) and recent deep-learning (DL) algorithms have been used with databases from different languages on supervised and unsupervised classification tasks [13]. However, automatization of emotion recognition is still challenging considering the significant number of complex parameters determining the accuracy and generalizability of computational methods.

Research suggests that the success of supervised ML and DL algorithms is contingent on the variation of emotional models (i.e., discrete vs. dimensional), dataset types (i.e., acted, elicited, natural or semi-natural), data pre-processing (i.e., framing, windowing, voice activity detection, normalization, noise reduction, feature selection) and supporting modalities (e.g., visual and physiological signals) [13,14]. Crucially, the extracted features constitute the core of model training process. These features are composed of physical acoustic parameters (i.e., Low Level Descriptors (LLDs)) such as prosodic and spectral features. In the past, several acoustic parameter feature sets were developed, which are now being used with linear/non-linear ML classifiers and complex Deep Neural Networks. For instance, Support Vector Machine (SVM), Random Forest (RF), k-Nearest Neighbors (KNN), Decision Tree (DT) (i.e., “C4.5”, see [15]), Linear Regression and Naïve-Bayes [16,17,18,19,20] are among the most popular classical machine-learning algorithms usually showing good overall accuracy within the databases with relatively low computational costs. These algorithms contribute to the aim of real-time raw emotion recognition in speech. Furthermore, recent developments provide us with complex artificial and deep neural network algorithms such as the Multilayer Perceptron Neural Network (MLP), Convolutional Neural Networks (CNN) and Recurrent Neural Networks (RNN) [21]. These complex algorithms are capable of handling challenging tasks although they require very high computing power, expensive hardware, and a significant amount of energy. Finally, we note that Hidden Markov Models (HMMs) are also successfully used for automatic emotion recognition [22] and were also recently used in combination with MFCCs to address other challenging tasks of automatic classification of complex non-vocal human sounds [23]. However, HMMs also require substantial resources in terms of computing power and time, which can be a limitation to their feasibility [24]. Overall, the work towards determining the best methods for VER remains ongoing.

In experimental studies on ML algorithm comparisons, there is still no consensus for which physical acoustic parameters are optimal for the highest recognition accuracy [14]. From the broadest perspective, extracted LLDs are listed under four main categories [13,25]: (a) Prosodic Features (i.e., Fundamental frequency/pitch (*F*_0_), energy-volume/intensity, duration/speech rate), (b) Spectral Features (e.g., Mel Frequency Cepstral Coefficients (MFCC), Linear Prediction Cepstral Coefficients (LPCC)), (c) Voice Quality Features: (e.g., Jitter, Shimmer and Harmonics-to-Noise Ratio-HNR) and d) Teager Energy Operator (TEO) Based Features. Among these features, a wide range of extraction methods were applied such as calculating global statistics (e.g., mean and range) or voiced region local level analyses of the *F*_0_ separately or in combination [26]. This variation in the feature extraction can also be seen in the MFCC features, which are among the most frequently used spectral features. For instance, the number of used MFCCs and applied statistical functionals varies across established default feature sets [27,28] (e.g., 12 MFCCs/3 Inter-Quartile Ranges–*emobase* or 1–4 MFCCs/20th to 80th percentile–*eGeMAPS*). With regard to these variations in feature extraction, further exploratory comparisons are needed.

During the past decades, several feature sets were developed combining some of these LLDs. Popular examples are emobase [27], GeMAPS, eGeMAPS [28] and IS-09 [29]. The feature extraction tool openSMILE may be the most popular software deriving the above listed acoustic parameters—involving long-term (i.e., global) features and short-term (i.e., local) stationary features. In particular, the latter allows defining temporal changes along chunks of speech signal [27]. Although feature sets have commonalities, they provide different levels of performances depending on the LLDs, statistical derivations but also the number of the extracted LLDs. In this manner, the effects of extracted features to the performance of ML classifiers needs to be investigated and cross-validated.

Considering these parameters in VER, the aim of this methodological study is to compare existing algorithms in order to identify relatively more accurate ML classifiers and physical acoustic parameters among some of the existing materials. It would also be crucial to identify the level of performance on each emotion class separately. Overall, the results would indicate the crucial parameters determining discrete emotion prediction accuracy.

## 2. Related Works

In the past, several studies conducted comparisons between combinations of several features and classifiers on the widely used Berlin Database of Emotional Speech (EMO-DB) [30] and more recent databases such as the Ryerson Audio-Visual Database of Emotional Speech and Song (RAVDESS) [31].

In the comparison of classical ML algorithms, the SVM and the KNN algorithms are frequently used. For example, SVM, KNN, Linear Discriminant Analysis (LDA) and Regularized Discriminant Analysis (RDA) were compared with a fusion of spectral and prosodic features on the EMO-DB and a Spanish emotional database [32]. Overall, results indicated accuracies between 62% and 81%, with the RDA performing best among these classifiers. In another study, the SVM outperformed KNN and Naïve-Bayes algorithms, showing up to 86% accuracy on the EMO-DB with a gain-ratio feature selection approach [19]. In a more recent study, Logistic Regression was reported to give better (in fact, ceiling, at 100%) performance compared to MLP (84.62%) and SVM (91,67%) algorithms on an unstandardized own database with MFCCs [33]. In these experimental designs, it is apparent that researchers used different kinds of feature extraction methods but also only a single set of features across the algorithms. In relation to this limitation, Sugan and colleagues [34] compared the SVM and the Feedforward Backpropagation Artificial Neural Network (FF-BP-ANN) across three different sets of cepstral features on the EMO-DB. However, this study contains no prosodic features as some other studies do (e.g., [35]), despite the distinctive characteristic effect of intonation and rhythm on vocal emotion expression [36].

Considering this comparison approach and its limitations in the literature, the aim of this study is to evaluate performance of ML algorithms across different feature sets containing different types of physical features (e.g., prosodic, spectral, voice quality) on the most frequently used EMO-DB database. This approach would provide a broader perspective to evaluate the performance of ML algorithms and their large variations of performance. In addition, we conduct statistical significance tests on the algorithm comparisons which are not found in previous research. Finally, another aim of this study is to discuss classification performances on each emotion class level in more detail, rather than focusing only on overall accuracy percentages.

## 3. Materials and Methods

### 3.1. Database

The Berlin Database of Emotional Speech (EMO-DB) [30] was used for the classification of the emotional states anger, boredom, disgust, fear, happiness, sadness and neutral. The EMO-DB comprises 10 German everyday life sentences (i.e., 5 short and 5 long sentences) that were recorded from 10 actors (5 females, 5 males) for each emotional state. In total, 535 utterances (Table 1) are used in the final form of the database (i.e., 127 anger, 69 fear, 46 disgust, 62 sadness, 71 happiness, 79 neutral, 81 boredom).

### 3.2. Feature Extraction

In total, 4 distinct feature sets of the openSMILE (Version 3.0) [27] were used for the extraction of physical acoustic parameters (also called Low-Level-Descriptors (LLDs)) and their statistical derivations: The openSMILE/openEAR “emobase” Feature Set (emobase), The INTERSPEECH 2009 Emotion Challenge Feature Set (IS-09) [29], The Geneva Minimalistic Acoustic Parameter Set (GeMAPS) and its extended version (eGeMAPS) [28].

The emobase feature set is composed of 988 attributes derived from 26 LLDs and their delta coefficients by implementing several statistical functions (Table 2) such as standard deviation, skewness, kurtosis, range and arithmetic mean. Included LLDs are Fundamental Frequency (*F*_0_), 12 MFCCs, Zero-Crossing Rate (ZCR), Probability of Voicing, Intensity, Loudness, *F*_0_ Envelope and 8 Line Spectral Frequencies.

The IS-09 contains 16 LLDs (Table 2) and 384 attributes. Fundamental Frequency (*F*_0_), 12 MFCCs, Zero-Crossing Rate (ZCR) and Probability of Voicing are common LLDs just as emobase. In addition, it also contains Root-Mean-Square (RMS) Energy parameters. As in emobase, the delta coefficients are computed and 12 statistical functionals are implemented (i.e., mean, standard deviation, kurtosis, skewness, minimum and maximum value, relative position, range and two linear regression coefficients with their mean square error).

The more recently developed GeMAPS feature set contains 18 LLDs (Table 2) including Fundamental Frequency (*F*_0_), H1-H2 Harmonic Difference (*F*_0_), H1-A3 Harmonic Difference (*F*_0_ − A3), Jitter, Formant 1-2-3 Frequency, Formant 1, Shimmer, Loudness, Harmonics to Noise Ratio (HNR), Alpha Ratio, Hammarberg Index, Spectral Slope 0–500 Hz and 500–100 Hz and Formant 1-2-3 Relative Energy. Mean and Coefficient of Variation are calculated on all smoothed LLDs. The 20th, 50th and 80th Percentile, the Range of 20th to 80th percentile, Mean and Standard Deviation of the Slope of rising/falling signal parts are applied only to pitch and loudness. In addition, several temporal features are added such as rate of loudness peaks and mean length of unvoiced regions (for more details see [25]). The extended version (eGeMAPS) contains additional spectral parameters MFCCs 1–4, Spectral flux and Formant 2–3 Bandwidth. In addition, Equivalent Sound Level, Voiced and unvoiced region inclusions are added to the eGeMAPS (Table 2).

### 3.3. Classifiers

In terms of the determining emotional classifiers, classifications were conducted with the Python wrapper package [37] of the WEKA [38] ML classifiers: Multilayer Perceptron Neural Network (MLP), Support Vector Machine with Sequential Minimal Optimization (SMO), J48 Decision Tree (DT), Random Forest (RF), k-Nearest Neighbor (KNN), Simple Logistic Regression (LOG) and Multinomial Logistic Regression (MLR). Classifier configurations were set to default values of the WEKA except the MLP.

The MLP contained 3 hidden layers. The number of nodes at the first layer varied depending on the number of input attributes (i.e., sum of the number of attributes and number of classes is divided by 2). The second and third hidden layers contained 32 and 16 nodes, respectively. Further neural network configurations were default values of the WEKA algorithm (i.e., learning rate = 0.3, momentum rate of the backpropagation = 0.2, number of epochs = 500).

### 3.4. Statistical Analyses

**Figure 1:**
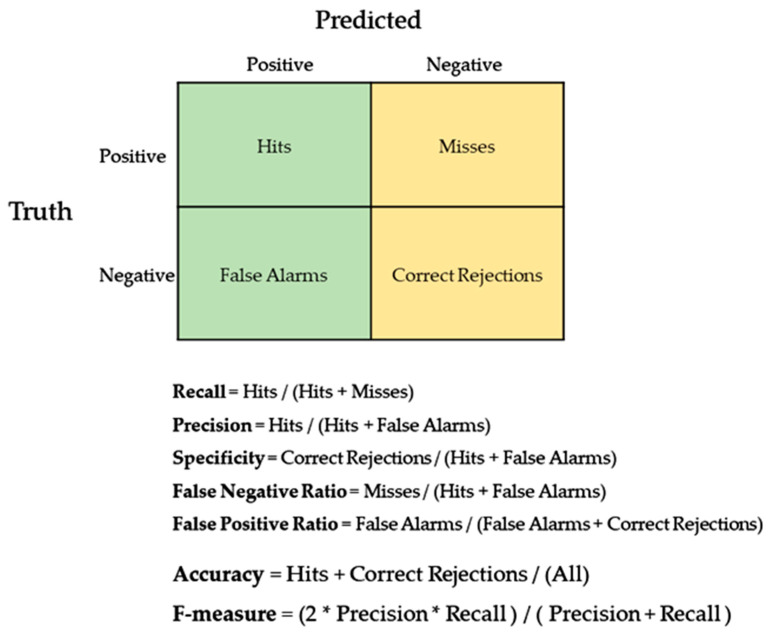
Computations for the prediction performance evaluations.

The overall performance matrix was based on overall accuracy (ACC) and F-measures (F) that calculated via precision and recall scores for each classifier and feature set combination (Figure 1).

Several model fit measures and weighted (w) averages along imbalanced classes of the database were calculated for the comparison of the models such as precision, recall, Area Under Precision Recall Curve (AUPRC), F-measure, Matthews Correlation Coefficient (MCC) [39], Area Under Curve (AUC—calculated via Receiver Operating Characteristics) [40], Cohen´s Kappa (κ) [41], and Root Mean Squared Error (RMSE).

In addition, significance tests were conducted on the accuracy percentages of the classifiers taking the best classifier as a test base and implementing 10-fold cross-validated paired T-tests. Comparisons were conducted on the final average accuracy of all 10-fold.

## 4. Results

### 4.1. 10-Fold Cross-Validation

**Figure 2:**
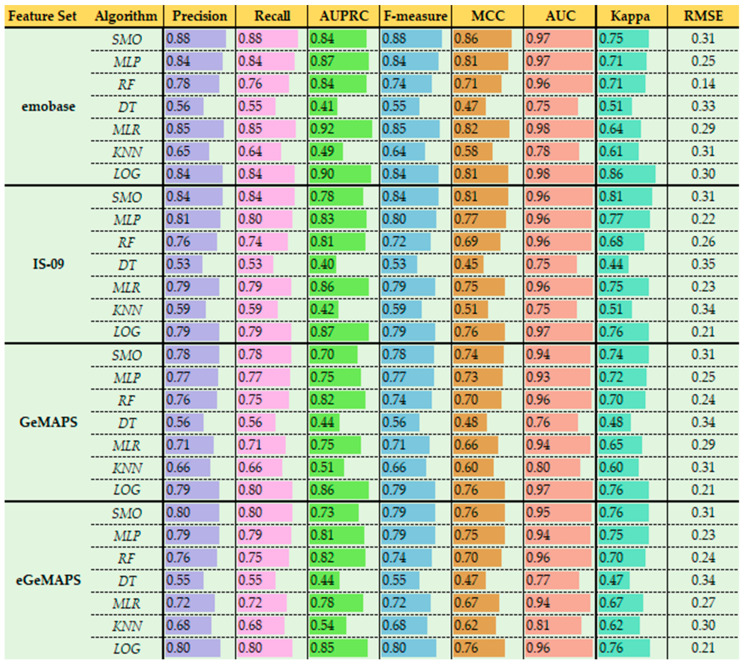
Classification performance measures among feature sets. Precision, Recall, AUPRC and AUC values are weighted averages among number of instances of each class in the database. Data bars represent values between 0 and 1. Length of the data bars are determined by the number in each cell.

In total, 4 feature sets and 7 classifiers were included in the 10-fold cross-validation studies on the EMO-DB (see also Figure S1 for all prediction performance evaluations in the supplementary materials). Overall, the most accurate model and feature set combination was the SMO classifier and the emobase feature set (ACC = 87.85%; AUPRC_w_ = 0.84; F_w_ = 0.88; MCC = 0.86; AUC_w_ = 0.97; κ = 75; RMSE = 0.31; see Table 3 and Figure 2). On all feature sets, SMO_acc range_ [78.32%: 87.85%], MLP_acc range_ [76.63%: 84.00%] and LOG_acc range_ [79.44%: 83.74%] showed relatively better performance compared to the decision tree-based DT, RF and KNN classifiers. Additional classification performance measures were also consistent in regard to this difference between two group of algorithms (Figure 2). Crucially, the MLR classifier reached up to 84.70% accuracy with the emobase feature set (AUPRC_w_ = 0.92; F_w_ = 0.85; MCC = 0.82; AUC_w_ = 0.98; κ = 0.64; RMSE = 0.29) while showing relatively low performance on the GeMAPS (ACC = 70.65%; AUPRC_w_ = 0.75; F_w_ = 0.71; MCC = 0.66; AUC_w_ = 0.94; κ = 0.65; RMSE = 0.29) and eGeMAPS (ACC = 71.96%; AUPRC_w_ = 0.78; F_w_ = 0.72; MCC = 0.67; AUC_w_ = 0.94; κ = 0.67; RMSE = 0.27) feature sets.

Overall, the worst accuracy performances were detected on DT _acc range_ [53.08%: 56.10%] and KNN _acc range_ [58.69%: 68.22%] classifiers. Although RF _acc range_ [73.46%: 75.70%] provided a substantial improvement compared to DT, it showed inferior performance compared to MLP, LOG and MLR, especially with emobase and IS-09 feature sets.

The feature sets emobase and IS-09 showed relatively better performance compared to the GeMAPS and eGeMAPS (Table 3). However, GeMAPS and eGeMAPS reached around 76% to 79% accuracy with the MLP, SMO and LOG classifiers. Specifically, with the MLR classifier, the performance difference was biggest between emobase (ACC = 84.70%; AUPRC_w_ = 0.92; F_w_ = 0.85; MCC = 0.82; AUC_w_ = 0.98; κ = 0.64; RMSE = 0.29) and GeMAPS (ACC = 70.65%; AUPRC_w_ = 0.75; F_w_ = 0.71; MCC = 0.66; AUC_w_ = 0.94; κ = 0.65; RMSE = 0.29) or eGeMAPS (ACC = 71.96%; AUPRC_w_ = 0.78; F_w_ = 0.72; MCC = 0.67; AUC_w_ = 0.94; κ = 0.67; RMSE = 0.27) feature sets.

Cross-validated paired *t*-tests also confirmed that the SMO classifier showed an overall better performance compared to most of the classifiers (Table 4). Particularly, SMO significantly outperformed DT, RF and KNN algorithms among all comparisons (although some of these differences were less prominent with the GeMAPS and eGeMAPS feature sets). Interestingly, the SMO classifier was significantly better than the LOG only on the emobase feature set (t = 4.56, *p* = 0.001). This indicates that the largest feature set emobase created a more robust difference between SMO and LOG.

Finally, comparisons of the SMO with the other two best classifiers MLR and MLP indicated that SMO slightly outperformed these. However, the difference to MLR was statistically significant only with the emobase (t = 3.58, *p* = 0.005) and GeMAPS (t = 3.81, *p* = 0.004) feature sets. With the similar pattern, SMO performed better than MLP only with the emobase (t = 4.79, *p* = 0.001), GeMAPS (t = 2.45, *p* = 0.014) and IS-09 feature sets (t = 3.05, *p* = 0.001).

### 4.2. F-Measures of Class Predictions and Confusion Matrices

**Figure 3:**
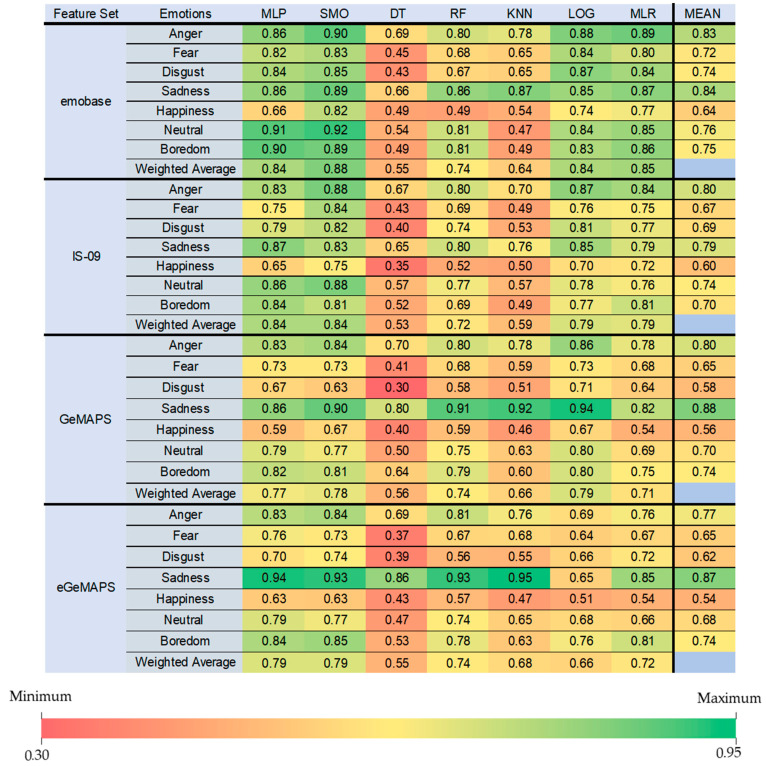
F-measures for each emotion. Color coding indicates performance, with dark green indicating best and dark red indicating poorest performance, and with yellow indicating intermediate classification performance, as shown in the color bar.

F-measures of each emotion class are displayed across classifiers and feature sets (Figure 3). Overall, the emotion class with the best prediction performance was “Sadness” _Fmean_[0.79:0.88] while the lowest performance was detected for “Happiness” _Fmean_[0.54:0.64].

Among the least accurate classifiers, it is apparent that F-measures of the “Sadness” class are still acceptable such as for RF [emobase = 0.86, IS-09 = 0.80, GeMAPS = 0.91, eGeMAPS = 0.93] and KNN [emobase = 0.87, IS-09 = 0.76, GeMAPS = 0.92, eGeMAPS = 0.95]. Crucially, this performance increase can be detected more correctly in GeMAPS and eGeMAPS sets when compared to emobase and IS-09 feature sets. Moreover, the least accurate classifier DT showed even good performance on the “Sadness” emotion, especially on GeMAPS (0.80) and eGeMAPS (0.86) feature sets. However, this increase in performance was not as prominent on emobase (0.66) and IS-09 (0.65) feature sets.

Conversely, even the most accurate classifiers showed lowest F-measures for the “Happiness” emotion such as MLP [emobase = 0.66, IS-09 = 0.65, GeMAPS = 0.59, eGeMAPS = 0.63], LOG [emobase = 0.74, IS-09 = 0.70, GeMAPS = 0.67, eGeMAPS = 0.51], SMO [emobase = 0.82, IS-09 = 0.75, GeMAPS = 0.67, eGeMAPS = 0.63] and MLR [emobase = 0.77, IS-09 = 0.72, GeMAPS = 0.54, eGeMAPS = 0.54]. However, it is crucial to note that this performance decrease of “Happiness” class detection among SMO and MLR algorithms was detected more prominently on GeMAPS and eGeMAPS feature sets compared to emobase and IS-09.

**Figure 4:**
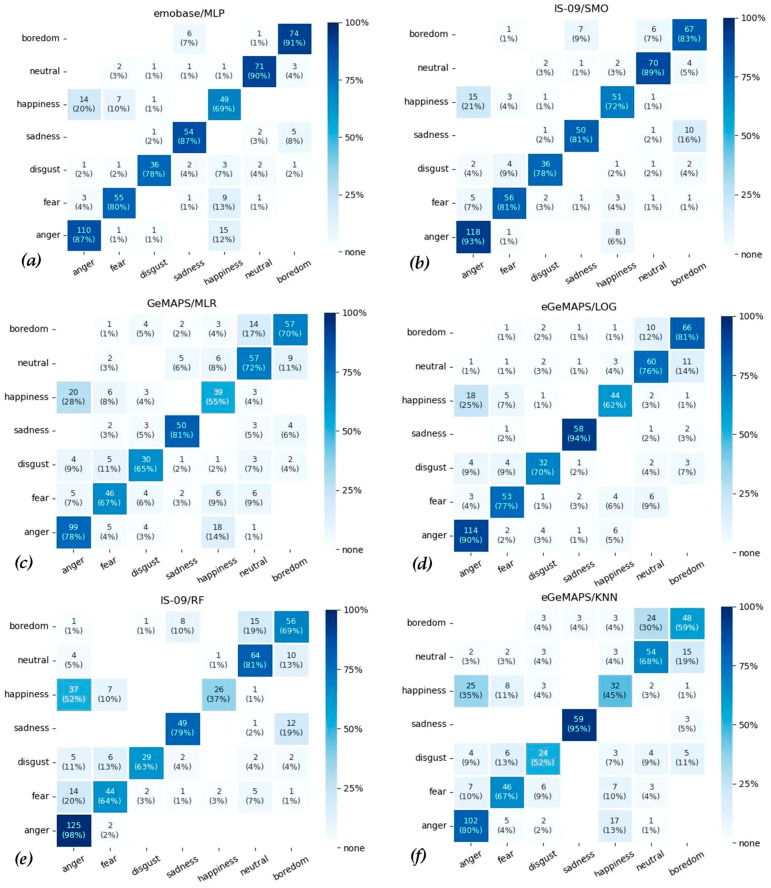
Confusion Matrices of the Predictions With (**a**) emobase/MLP, (**b**) IS-09/SMO, (**c**) GeMAPS/MLR, (**d**) eGeMAPS/LOG, (**e**) IS-09/RF, (**f**) eGeMAPS/KNN. The *x*-axis represents the ground truth labels and the *y*-axis represents predicted labels. Note: Figures give percentages determining the color map but also provide absolute numbers in parentheses to transparently indicate different base frequencies of the predicted emotions. Note also that percentages and numbers are omitted for empty cells to enhance readability.

Furthermore, confusion matrices for each classifier and feature set combinations were extracted (Figure 4 and see Figure S2 for all in the supplementary materials). As a complementary finding, some of the confusion matrices seem problematic in regard to the low performance of the class “Happiness” predictions. For instance, 20% (14 out of 71) of “Happiness” voices were classified as “Anger” and 12% (15 out of 127) of “Anger” voices were classified as “Happiness” by the MLP classifier with the emobase feature set (Figure 4a). Similarly, these false classifications were 21% (15/71) “Anger” and 6% (8/127) “Happiness” on the SMO classifier with IS-09 feature set (Figure 4b). Furthermore, GeMAPS and eGeMAPS feature sets were also problematic in terms of the differentiation of “Happiness” and “Anger”, for instance with MLR and LOG classifiers (Figure 4c,d). Moreover, this problem is more apparent among the classifiers with overall low performance such as RF with IS-09 and KNN with eGeMAPS (Figure 4e,f), showing 52% (37/71) and 35% (25/71) of confusion, respectively.

Finally, some confusion matrices also indicate a differentiation problem between the “Boredom” and “Neutral” classes. These cases were observed especially among the classifications with low overall performance such as RF with IS-09 (Figure 4e), KNN with eGeMAPS (Figure 4f), KNN with emobase, DT with GeMAPS and eGeMAPS (please see supplementary materials Figure S2).

## 5. Discussion

Conducting successful automatic vocal emotion recognition (VER) in speech is challenging and depends on numerous complex factors. With a set of analyses, we aimed to identify the ML algorithms and feature sets with best performances on the EMO-DB emotional speech database. Performance analyses on 10-fold cross-validation indicated that SMO, MLP and LOG showed more accurate predictions compared to DT, RF and KNN. Overall, the classifications were more accurate with the emobase and IS-09 feature sets compared to GeMAPS and eGeMAPS. It is crucial to better understand these differences.

The SMO algorithm showed the best overall accuracy percentage, achieving 87.9% with the emobase feature set in our analyses and comparisons. Further, our SMO results indicate relatively better overall accuracy compared to classical SVM, which has been widely used in the vocal emotion recognition literature [26,42,43]. The Sequential Minimal Optimization algorithm in our analyses provided significant improvements, specifically with the implemented default feature sets.

Considering the classifications with low overall accuracy in our analyses, it is important to note that decision-tree-based algorithms (i.e., DT and RF) are not ideal for the used emotional dataset and acoustic parameter sets, at least when it comes to the default configurations of WEKA. Even though accuracy was low in our C4.5 classical DT algorithm, it should be noted that the decision-tree concept seems to be successfully implemented in several other algorithms. For instance, Lee et al. [18] managed to reach up to 89.6% accuracy by implementing the Extreme Learning Machine (ELM) technique and the SVM binary decision-tree (DTSVM) algorithm with a correlational feature selection approach. Moreover, in a study with a new approach called DNN-decision tree, SVM algorithm reached 75.8% accuracy on the EMO-DB database [44]. However, the feature extraction methods are not identical among these studies, which makes the performance comparison difficult.

KNN was another algorithm with relatively low overall accuracies in our study. This outcome stands in contrast to previous research that provided very high overall accuracies with the KNN algorithm on VER. This discrepancy could potentially also relate to differences in the applied feature extraction method. For instance, Khan et al. [45] reported reaching 91.7% overall accuracy on an own database using the Forward Selection (FS) feature selection method. Using the EMO-DB database, Zhu and Ahamd [46] showed that the KNN algorithm reached to up to 78.8% accuracy by using Gamma Frequency Cepstral Frequencies (GTCCs) in addition to prosodic features and spectral frequencies.

All this suggests that the performance of the implemented algorithms is highly dependent on the acoustic parameters used as input set to the models. Accordingly, further refinements in this regard seem a promising way to enhance accuracy.

Interestingly, more contemporary default feature sets as implemented in openSMILE (i.e., GeMAPS and eGeMAPS) failed to outperform the older and more extensive feature sets. This difference might be mainly based on the size of the feature sets. Note that the GeMAPS feature set actually could have an advantage in the real-life task as the larger feature sets could have a problem of overfitting to the training set [28]. In addition, the smaller feature set of the GeMAPS should be advantageous in real-time applications. In this manner, it would be even more informative to conduct feature set and algorithm comparisons on unseen data from other databases or real life, rather than just from untrained data within the same dataset.

As a limitation of the present study, note that we conducted analyses only on the EMO-DB database. Although this is one of the most popular databases in the literature, it has a relatively small number of samples (i.e., 535) compared to other widely used databases such as the Ryerson Audio-Visual Database of Emotional Speech and Song (RAVDESS; 2496 samples) [31] or the Interactive Emotional Dyadic Motion Capture Database (IEMOCAP; 1150 samples) [47]. It is important to appreciate differences between these databases in terms of producing emotional expressions (e.g., posing, acting to script, mood induction, interaction vs. solo expression) or variability of utterances (e.g., two sentences in RAVDESS, 10 sentences in EMO-DB and open spontaneous material in IEMOCAP). In addition, databases differ with respect to the underlying emotion model, number of emotion categories or dimensions, and language. In this manner, future research in this area could reduce database dependency by using multiple databases with different characteristics. At the same time, reviewing databases with different characteristics could help to select the best material for a particular research question. For instance, databases that use mood induction could have advantages over databases that use enacted/posed emotions when the aim is to assess algorithms’ performance in the context of real-life emotions. Moreover, whereas existing databases tend to focus on salient and basic emotions, the increasing trend in emotion research to study more subtle emotions (e.g., pride, compassion, gratitude, admiration, desire; for review see [48]) could call for the development of databases that permit research on the automatic classification of subtle emotions.

Finally, the confusion matrices provide an informative pattern of misclassifications regarding specific emotion. Most prominently, nearly all classifications had a tendency for confusing “Happiness” and “Anger” categories, especially with the GeMAPS and eGeMAPS feature sets. This confusion could be related to the fact that both emotions are expressed with high arousal that results in physically high pitch and volume/intensity on the vocal samples [49]. In addition, the second most confused emotion categories were “Boredom” and “Neutral”. Interestingly, “Boredom” is the only emotion that is not one of the Ekman´s six basic emotion categories [50]. At the same time, in a 2D emotional model, specifically “indifferent boredom” has a rather neutral position in terms of valence [51] exhibiting low volume/intensity in relation to lower arousal level.

## 6. Conclusions

This paper has provided a comparison of classical ML algorithms for automatic vocal emotion recognition in speech, which recently gained importance in a growing range of applications. The present analyses were performed on existing recordings and feature sets of vocal emotions, and the findings therefore will be of interest for researchers who use these algorithms for off-line automatic emotion analysis. Human emotions are inherently multimodal in nature [52], such that sensor integration and simultaneous consideration of facial, vocal, and bodily data (where available) can be expected to enhance automatic emotion recognition. Where applications depend on a real-time automatic analysis of vocal emotions, more comparisons between efficient algorithms for real-time analyses will be warranted. At the same time, such work could benefit from considering the tight link between the perception and expression of human vocal emotions [53]. In the past two decades, substantial progress has been made in methods for automatic emotion recognition. To achieve large-scale usability in applied fields of diagnosis, intervention, communication research or human–robot interaction, both systematic evaluation of available algorithms and ongoing efforts at refining these are indispensable.

## Figures and Tables

**Table 1:** Number of instances for each emotion class of the EMO-DB.

Emotions	Number of Instances
Anger	127
Fear	69
Disgust	46
Sadness	62
Happiness	71
Boredom	81
Neutral	79
**Total**	**535**

**Table 2:** Low-Level-Descriptors and Functionals of Each Feature Set. (*) indicates features of emobase and IS-09 distinct from each other. (**) indicates the features of eGeMAPS in addition to GeMAPS features.

Feature Sets	Low-Level-Descriptors	Functionals
emobase and IS-09 (Common Features)	*F*_0_, 12 MFCCs, ZCR, Probability of Voicing	Mean, Standard Deviation, Skewness, Kurtosis, Minimum and Maximum Value, Range, Slope and Offset of Linear Approximation with Quadratic Error
emobase	* Intensity, Loudness, *F*_0_ Envelope, 8 Line Spectral Frequencies	* 3 Inter-Quartile Ranges, Quartile 1–3
IS-09	* (RMS) Energy	-
GeMAPS	*F*_0_, H1-H2 Harmonic Difference *F*_0_, H1-A3 Harmonic Difference (*F*_0_ − A3), Jitter, Formant 1-2-3 Frequency, Formant 1, Shimmer, Loudness, HNR, Alpha Ratio, Hammarberg Index, Spectral Slope 0–500 Hz and 500–100 Hz, Formant 1-2-3 Relative Energy	Mean, Coefficient of Variation; (For loudness and *F*_0_): *20th, 50th and 80th Percentile, the Range of 20th to 80th percentile, Mean and Standard* *Deviation of the Slope of rising/falling signal parts*; (6 Additional Temporal Features): *Rate of Loudness Peaks, Mean Length and Standard Deviation on the Regions F*_0_ *> 0 and F*_0_ *= 0, Pseudo Syllable Rate*
eGeMAPS	** MFCCs 1–4, Spectral Flux and Formant 2–3 Bandwidth	* Equivalent Sound Level. Voiced and unvoiced region inclusions vary among some LLDs.

**Table 3:** Accuracy percentages of each classifier on each feature set. Classifier and feature set names and abbreviations are written bold.

%	MLP	SMO	DT	RF	KNN	LOG	MLR
**emobase**	84.00	87.85	54.95	75.70	63.93	83.74	84.70
**IS-09**	80.37	83.74	53.08	73.46	58.69	79.44	78.51
**GeMAPS**	76.63	78.32	56.10	75.00	66.00	79.63	70.65
**eGeMAPS**	79.25	79.63	55.14	74.77	68.22	79.81	71.96

**Table 4:** Cross-Validated Paired T-Test Comparison (two-tailed) with the Test Base SMO Classifier. *: *p* < 0.05. **: *p* ≤ 0.001. Note that positive t-values indicate better performance of the SMO classifier. Classifier and feature set names and abbreviations are written bold.

Feature Set	MLP	DT	RF	KNN	LOG	MLR
**emobase**	*t* = 4.79	*t* = 14.78	*t* = 12.21	*t* = 10.93	*t* = 4.56	*t* = 3.58
	*p* = 0.001 **	*p* < 0.001 **	*p* < 0.001 **	*p* < 0.001 **	*p* = 0.001 **	*p* = 0.005 *
**IS-09**	*t* = 3.05	*t* = 10.00	*t* = 7.20	*t* = 10.77	*t* = 1.40	*t* = 1.74
	*p* = 0.001 **	*p* < 0.001 **	*p* < 0.001 **	*p* < 0.001 **	*p* = 0.198	*p* = 0.116
**GeMAPS**	*t* = 2.45	*t* = 17.24	*t* = 2.58	*t* = 5.04	*t* = −0.49	*t* = 3.81
	*p* = 0.014 *	*p* < 0.001 **	*p* = 0.030 *	*p* = 0.001 **	*p* = 0.638	*p* = 0.004 *
**eGeMAPS**	*t* = 1.12	*t* = 12.50	*t* = 1.58	*t* = 5.36	*t* = 1.04	*t* = 1.22
	*p* = 0.292	*p* < 0.001 **	*p* = 0.150	*p* = 0.001 **	*p* = 0.328	*p* = 0.254

## Data Availability

The implemented database (http://emodb.bilderbar.info/start.html accessed on 5 June 2022) and feature sets (https://github.com/audeering/opensmile/releases/tag/v3.0.0 accessed on 1 July 2022) are publicly available. The scripts in Python programming language, extracted data and raw outputs can be found via Open-Science-Framework (https://osf.io/k9usg/?view_only=d4b0ede657544849916e79a204956074).

## Supplementary Materials

The following supporting information can be downloaded at: https://www.mdpi.com/article/10.3390/s22197561/s1, Figure S1: Prediction Performance Evaluations; Figure S2: Confusion Matrices.
