# Supplementary material for: A Comparison of Machine Learning Algorithms and Feature Sets for Automatic Vocal Emotion Recognition in Speech
Source: Sensors (Basel). 2022 Oct 6;22(19):7561. doi: 10.3390/s22197561 (PMC9571288; doi:10.3390/s22197561)
Supplement: Supplementary file 1 [file sensors-22-07561-s001.zip › Figure S1.pdf]

Figure S1. Prediction Performance Evaluations

Note. The matrices are placed in the following order:

Page 2: emobase/DT, emobase/KNN, emobase/LOG, emobase/MLP, emobase/MLR, emobase/RF

Page 3: emobase/SMO, IS-09/DT, IS-09/KNN, IS-09/MLP, IS-09/LOG, IS-09/RF

Page 4: IS-09/MLR, IS-09/SMO, GeMAPS/RF, GeMAPS/MLR, GeMAPS/MLP, GeMAPS/DT

Page 5: GeMAPS/KNN, GeMAPS/SMO, eGeMAPS/RF, eGeMAPS/MLR, GeMAPS/LOG, eGeMAPS/DT,

Page 6: eGeMAPS/MLP, eGeMAPS/KNN, eGeMAPS/SMO

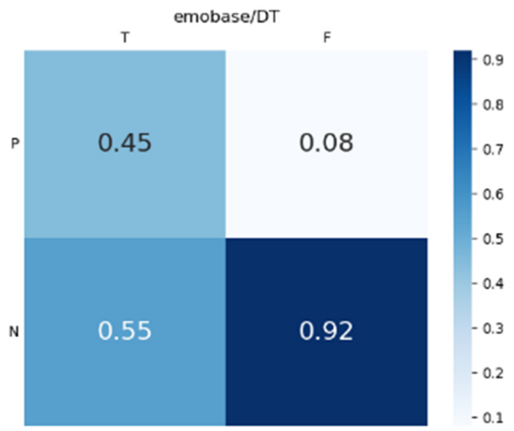
emobase/DT
T
F
P
0.45
0.08
N
0.55
0.92
0.9
0.8
0.7
0.6
0.5
0.4
0.3
0.2
0.1
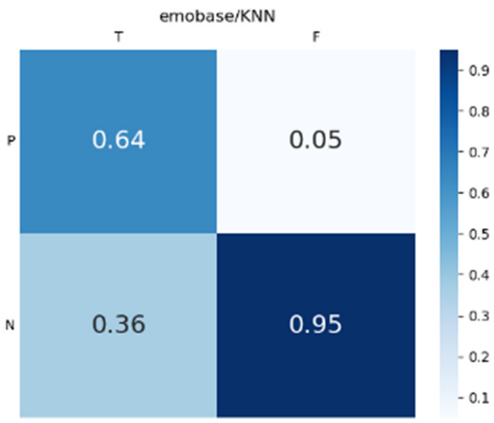
emobase/KNN
T
F
P
0.64
0.05
N
0.36
0.95
0.9
0.8
0.7
0.6
0.5
0.4
0.3
0.2
0.1
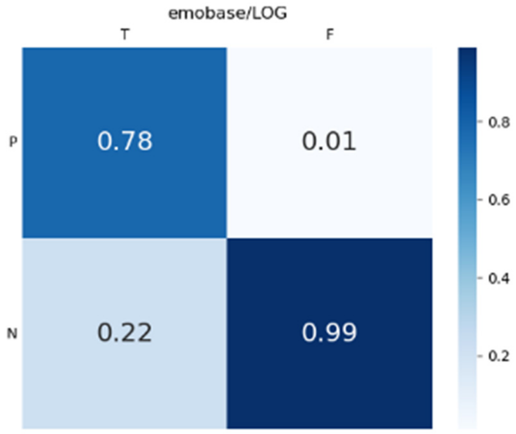
emobase/LOG
T
F
P
0.78
0.01
N
0.22
0.99
0.8
0.6
0.4
0.2
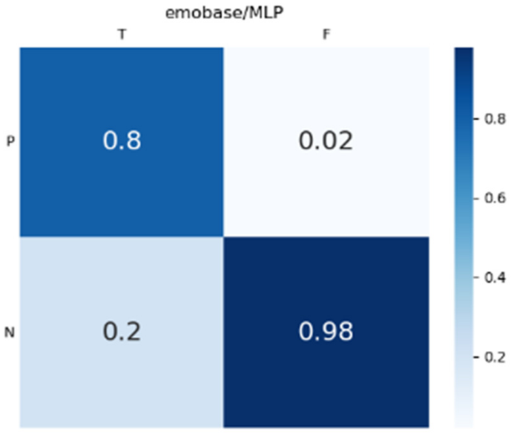
emobase/MLP
T
F
P
0.8
0.02
N
0.2
0.98
0.8
0.6
0.4
0.2
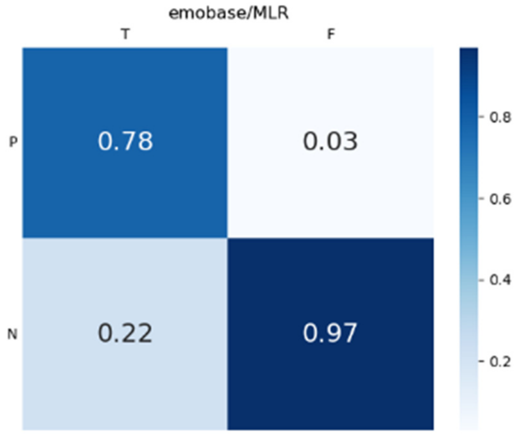
emobase/MLR
T
F
P
0.78
0.03
N
0.22
0.97
0.8
0.6
0.4
0.2
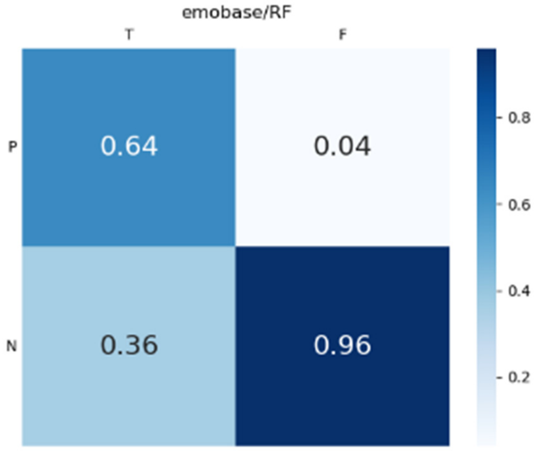
emobase/RF
T
F
P
0.64
0.04
N
0.36
0.96
0.8
0.6
0.4
0.2

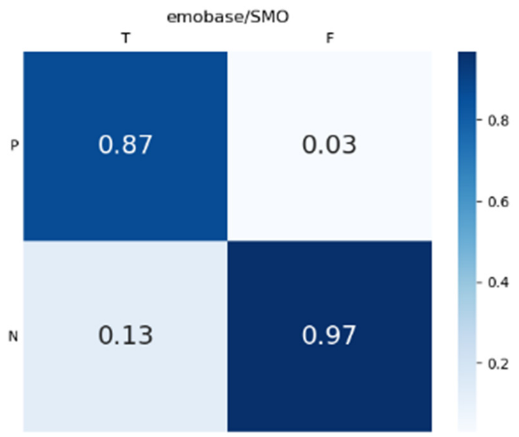
emobase/SMO
T
F
P
N
0.87
0.03
0.13
0.97

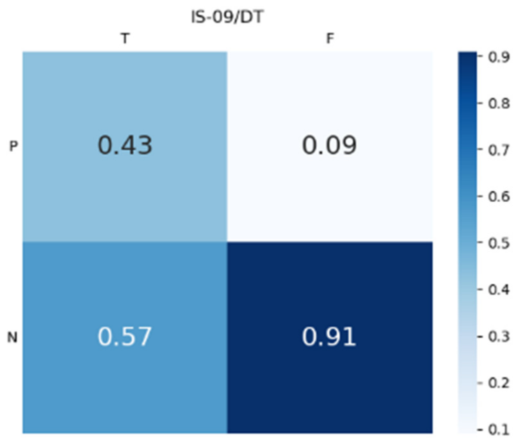
IS-09/DT
T
F
P
N
0.43
0.09
0.57
0.91

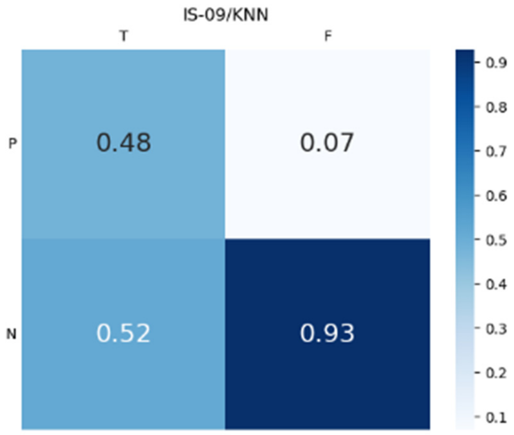
IS-09/KNN
T
F
P
N
0.48
0.07
0.52
0.93

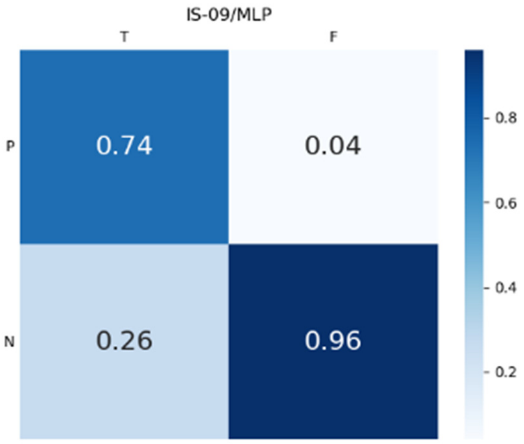
IS-09/MLP
T
F
P
N
0.74
0.04
0.26
0.96

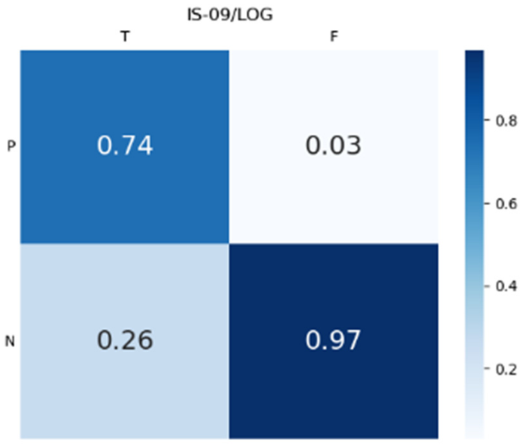
IS-09/LOG
T
F
P
N
0.74
0.03
0.26
0.97

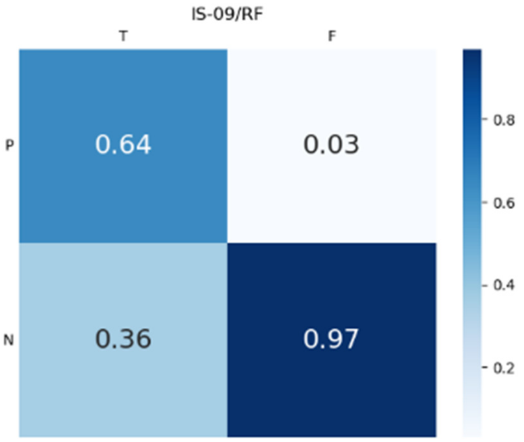
IS-09/RF
T
F
P
N
0.64
0.03
0.36
0.97

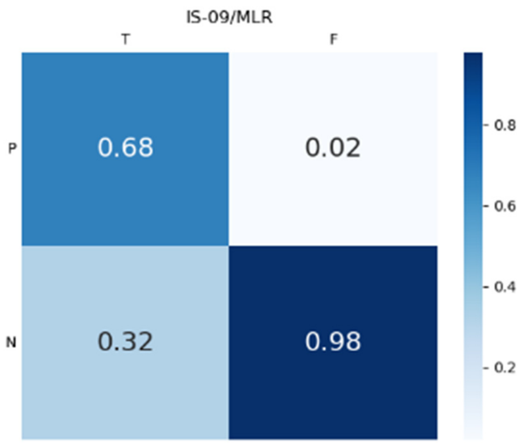
IS-09/MLR
T
F
P
N
0.68
0.02
0.32
0.98
0.8
0.6
0.4
0.2

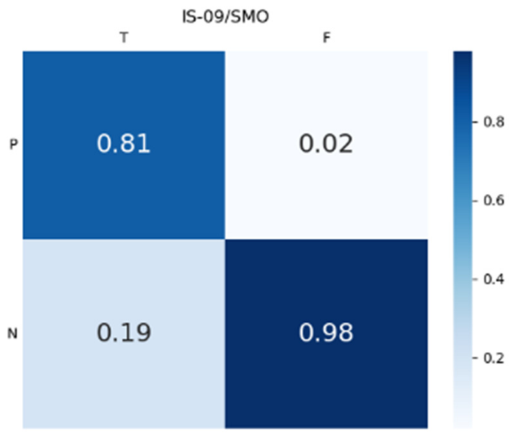
IS-09/SMO
T
F
P
N
0.81
0.02
0.19
0.98
0.8
0.6
0.4
0.2

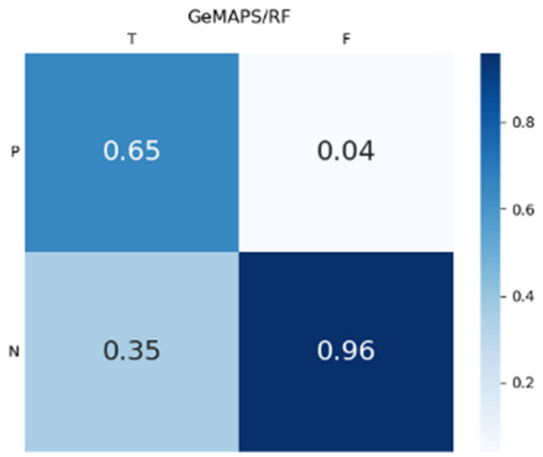
GeMAPS/RF
T
F
P
N
0.65
0.04
0.35
0.96
0.8
0.6
0.4
0.2

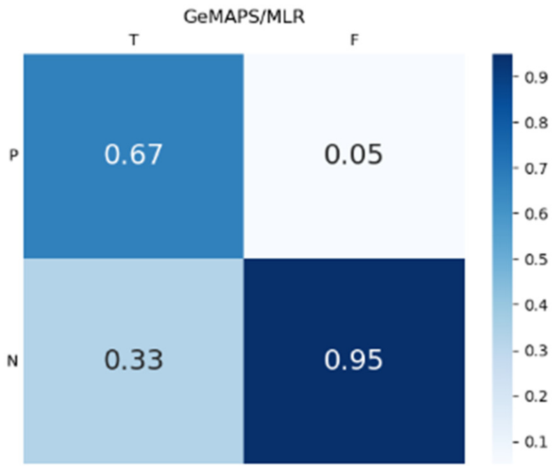
GeMAPS/MLR
T
F
P
N
0.67
0.05
0.33
0.95
0.9
0.8
0.7
0.6
0.5
0.4
0.3
0.2
0.1

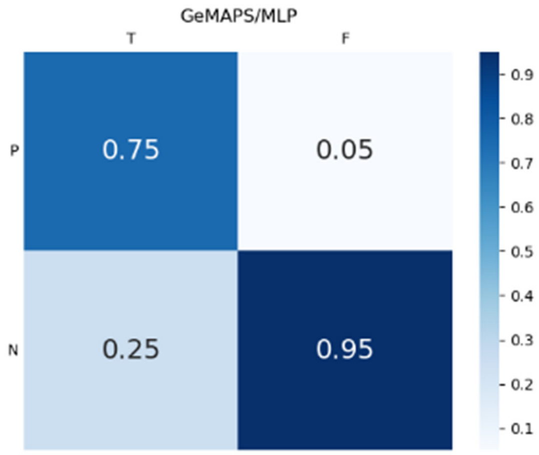
GeMAPS/MLP
T
F
P
N
0.75
0.05
0.25
0.95
0.9
0.8
0.7
0.6
0.5
0.4
0.3
0.2
0.1

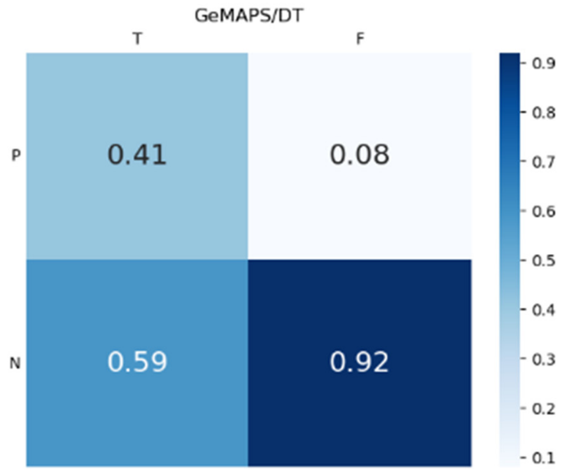
GeMAPS/DT
T
F
P
N
0.41
0.08
0.59
0.92
0.9
0.8
0.7
0.6
0.5
0.4
0.3
0.2
0.1

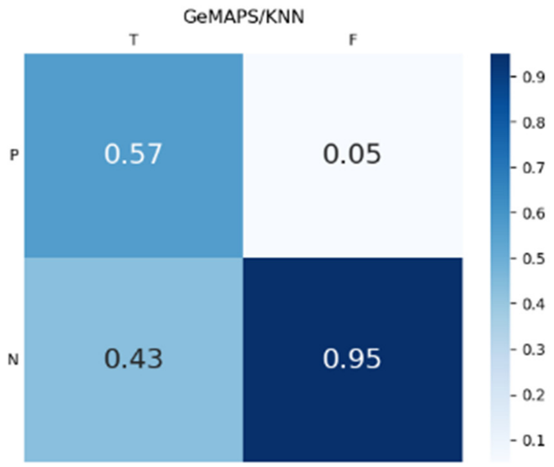
GeMAPS/KNN
T
F
P
N
0.57
0.05
0.43
0.95
0.9
0.8
0.7
0.6
0.5
0.4
0.3
0.2
0.1

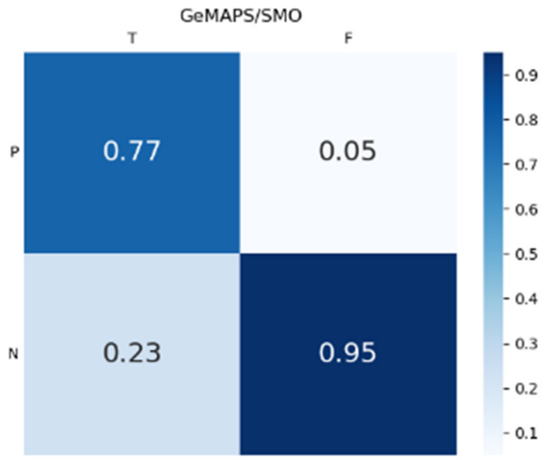
GeMAPS/SMO
T
F
P
N
0.77
0.05
0.23
0.95
0.9
0.8
0.7
0.6
0.5
0.4
0.3
0.2
0.1

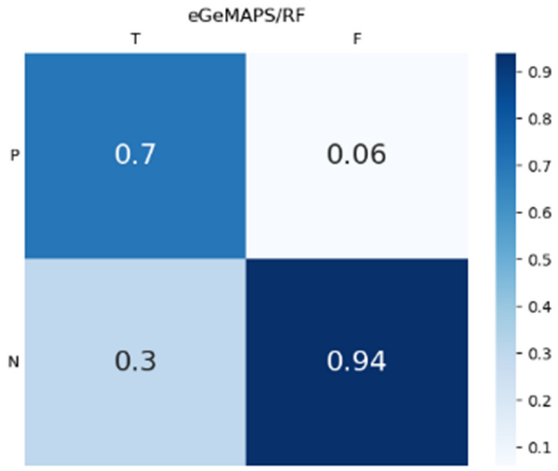
eGeMAPS/RF
T
F
P
N
0.7
0.06
0.3
0.94
0.9
0.8
0.7
0.6
0.5
0.4
0.3
0.2
0.1

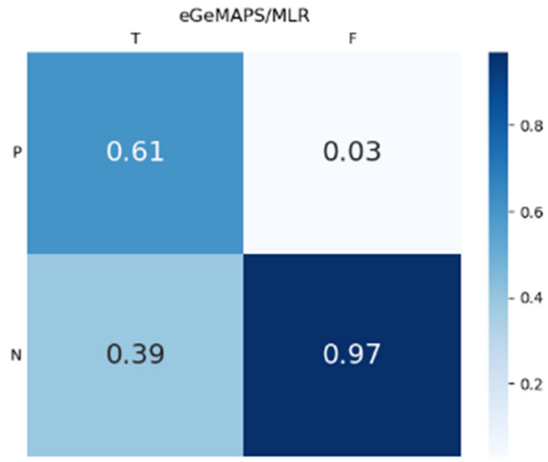
eGeMAPS/MLR
T
F
P
N
0.61
0.03
0.39
0.97
0.8
0.6
0.4
0.2

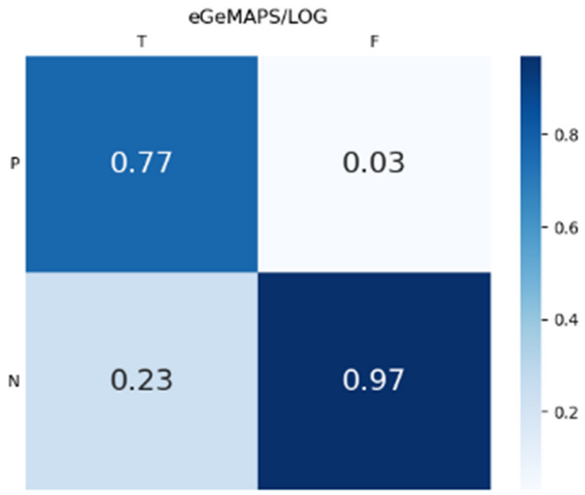
eGeMAPS/LOG
T
F
P
N
0.77
0.03
0.23
0.97
0.8
0.6
0.4
0.2

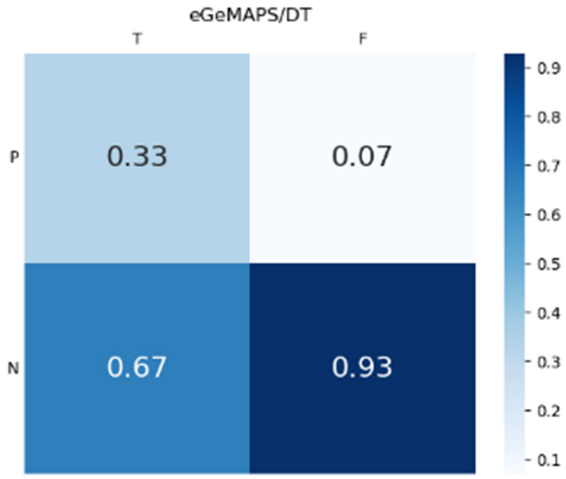
eGeMAPS/DT
T
F
P
N
0.33
0.07
0.67
0.93
0.9
0.8
0.7
0.6
0.5
0.4
0.3
0.2
0.1

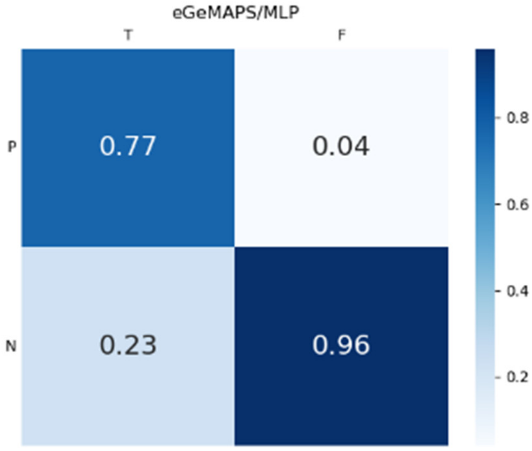
eGeMAPS/MLP
T
F
P
N
0.77
0.04
0.23
0.96
0.8
0.6
0.4
0.2

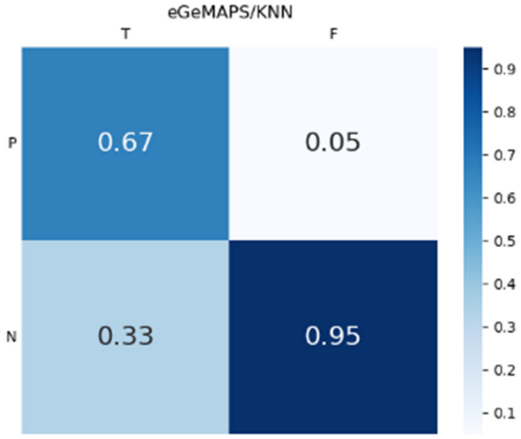
eGeMAPS/KNN
T
F
P
N
0.67
0.05
0.33
0.95
0.9
0.8
0.7
0.6
0.5
0.4
0.3
0.2
0.1

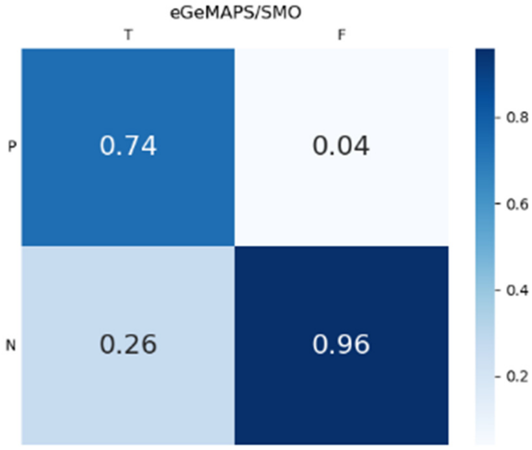
eGeMAPS/SMO
T
F
P
N
0.74
0.04
0.26
0.96
0.8
0.6
0.4
0.2
