# Supplementary material for: A Comparison of Machine Learning Algorithms and Feature Sets for Automatic Vocal Emotion Recognition in Speech
Source: Sensors (Basel). 2022 Oct 6;22(19):7561. doi: 10.3390/s22197561 (PMC9571288; doi:10.3390/s22197561)
Supplement: Supplementary file 1 [file sensors-22-07561-s001.zip › Figure S2.pdf]

Figure S2. Confusion Matrices

Note. The matrices are placed in the following order:

Page 2: emobase/DT, emobase/KNN, emobase/LOG, emobase/MLP, emobase/MLR, emobase/RF

Page 3: emobase/SMO, IS-09/DT, IS-09/KNN, IS-09/LOG, IS-09/MLP, IS-09/MLR

Page 4: IS-09/RF, IS-09/SMO, GeMAPS/DT, GeMAPS/RF, GeMAPS/MLR, GeMAPS/MLP

Page 5: GeMAPS/LOG, GeMAPS/KNN, GeMAPS/SMO, eGeMAPS/MLP, eGeMAPS/RF, eGeMAPS/MLR

Page 6: eGeMAPS/LOG, eGeMAPS/KNN, eGeMAPS/DT, eGeMAPS/SMO

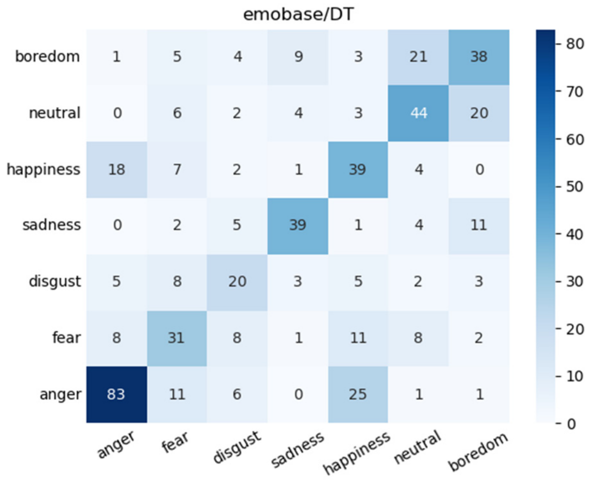
emobase/DT
boredom 1 5 4 9 3 21 38
neutral 0 6 2 4 3 44 20
happiness 18 7 2 1 39 4 0
sadness 0 2 5 39 1 4 11
disgust 5 8 20 3 5 2 3
fear 8 31 8 1 11 8 2
anger 83 11 6 0 25 1 1
anger fear disgust sadness happiness neutral boredom
80 70 60 50 40 30 20 10 0

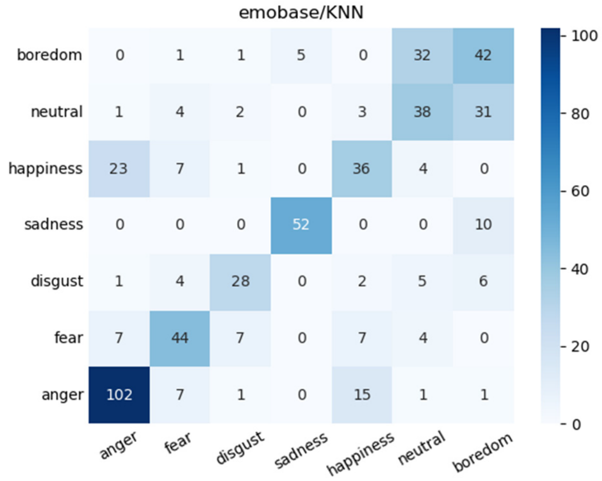
emobase/KNN
boredom 0 1 1 5 0 32 42
neutral 1 4 2 0 3 38 31
happiness 23 7 1 0 36 4 0
sadness 0 0 0 52 0 0 10
disgust 1 4 28 0 2 5 6
fear 7 44 7 0 7 4 0
anger 102 7 1 0 15 1 1
anger fear disgust sadness happiness neutral boredom
100 80 60 40 20 0

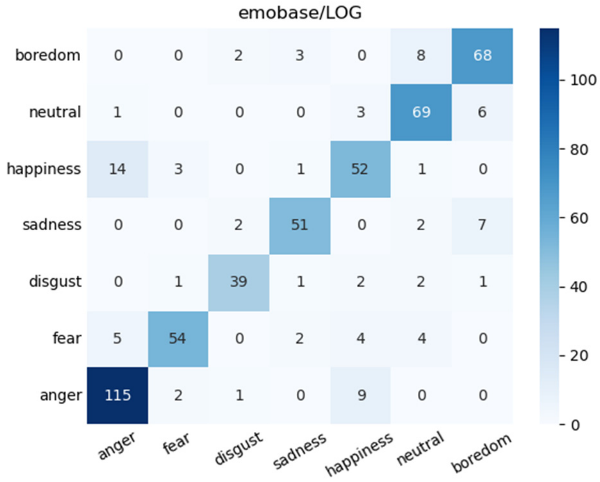
emobase/LOG
boredom 0 0 2 3 0 8 68
neutral 1 0 0 0 3 69 6
happiness 14 3 0 1 52 1 0
sadness 0 0 2 51 0 2 7
disgust 0 1 39 1 2 2 1
fear 5 54 0 2 4 4 0
anger 115 2 1 0 9 0 0
anger fear disgust sadness happiness neutral boredom
100 80 60 40 20 0

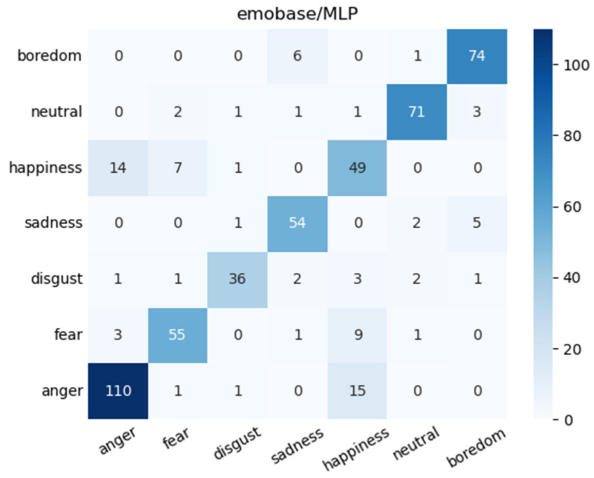
emobase/MLP
boredom 0 0 0 6 0 1 74
neutral 0 2 1 1 1 71 3
happiness 14 7 1 0 49 0 0
sadness 0 0 1 54 0 2 5
disgust 1 1 36 2 3 2 1
fear 3 55 0 1 9 1 0
anger 110 1 1 0 15 0 0
anger fear disgust sadness happiness neutral boredom
100 80 60 40 20 0

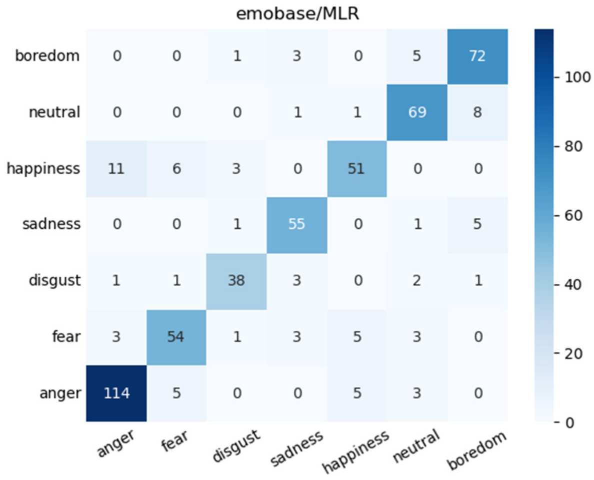
emobase/MLR
boredom 0 0 1 3 0 5 72
neutral 0 0 0 1 1 69 8
happiness 11 6 3 0 51 0 0
sadness 0 0 1 55 0 1 5
disgust 1 1 38 3 0 2 1
fear 3 54 1 3 5 3 0
anger 114 5 0 0 5 3 0
anger fear disgust sadness happiness neutral boredom
100 80 60 40 20 0

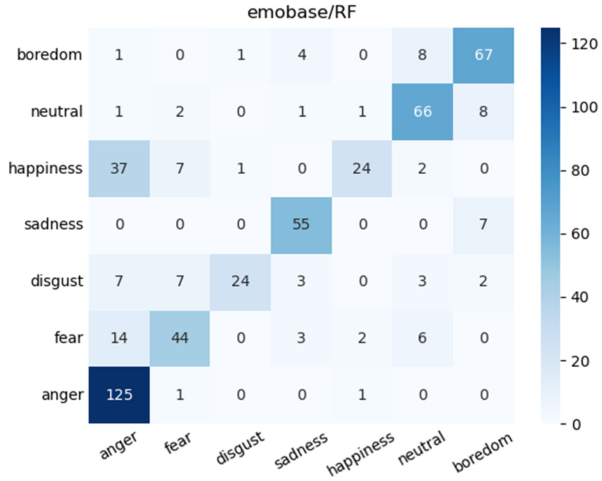
emobase/RF
boredom 1 0 1 4 0 8 67
neutral 1 2 0 1 1 66 8
happiness 37 7 1 0 24 2 0
sadness 0 0 0 55 0 0 7
disgust 7 7 24 3 0 3 2
fear 14 44 0 3 2 6 0
anger 125 1 0 0 1 0 0
anger fear disgust sadness happiness neutral boredom
120 100 80 60 40 20 0

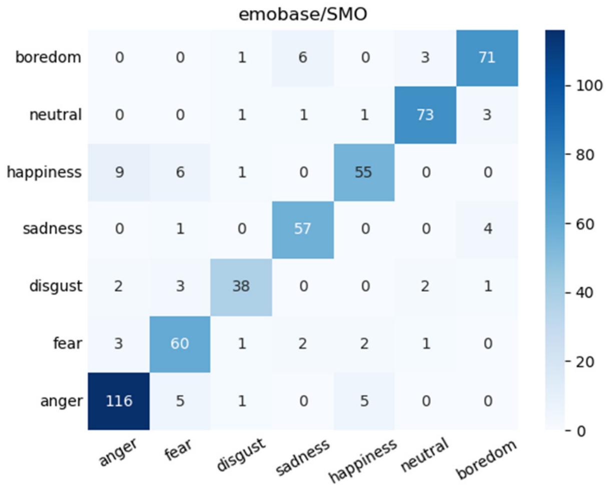
emobase/SMO
boredom 0 0 1 6 0 3 71
neutral 0 0 1 1 1 73 3
happiness 9 6 1 0 55 0 0
sadness 0 1 0 57 0 0 4
disgust 2 3 38 0 0 2 1
fear 3 60 1 2 2 1 0
anger 116 5 1 0 5 0 0
anger fear disgust sadness happiness neutral boredom
100 80 60 40 20 0

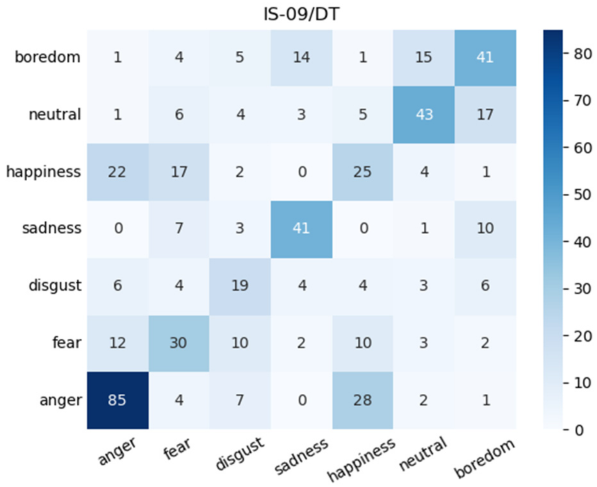
IS-09/DT
boredom 1 4 5 14 1 15 41
neutral 1 6 4 3 5 43 17
happiness 22 17 2 0 25 4 1
sadness 0 7 3 41 0 1 10
disgust 6 4 19 4 4 3 6
fear 12 30 10 2 10 3 2
anger 85 4 7 0 28 2 1
anger fear disgust sadness happiness neutral boredom
80 70 60 50 40 30 20 10 0

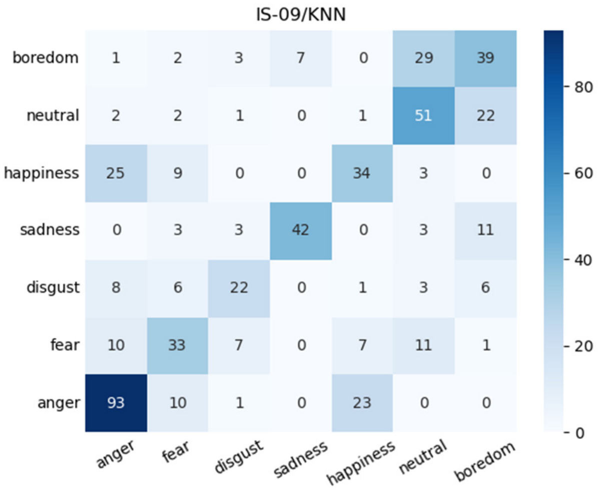
IS-09/KNN
boredom 1 2 3 7 0 29 39
neutral 2 2 1 0 1 51 22
happiness 25 9 0 0 34 3 0
sadness 0 3 3 42 0 3 11
disgust 8 6 22 0 1 3 6
fear 10 33 7 0 7 11 1
anger 93 10 1 0 23 0 0
anger fear disgust sadness happiness neutral boredom
80 60 40 20 0

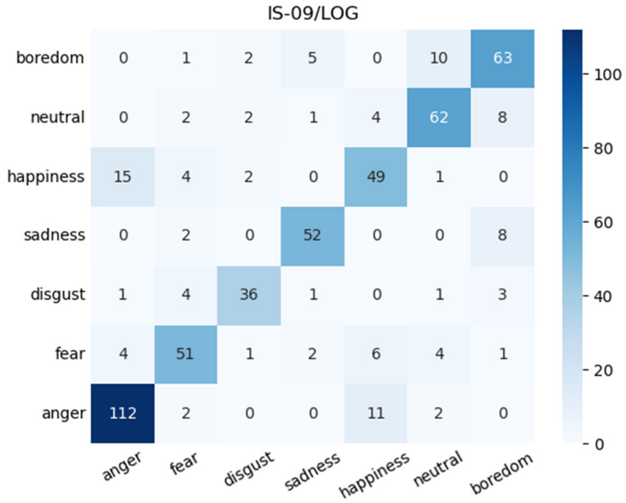
IS-09/LOG
boredom 0 1 2 5 0 10 63
neutral 0 2 2 1 4 62 8
happiness 15 4 2 0 49 1 0
sadness 0 2 0 52 0 0 8
disgust 1 4 36 1 0 1 3
fear 4 51 1 2 6 4 1
anger 112 2 0 0 11 2 0
anger fear disgust sadness happiness neutral boredom
100 80 60 40 20 0

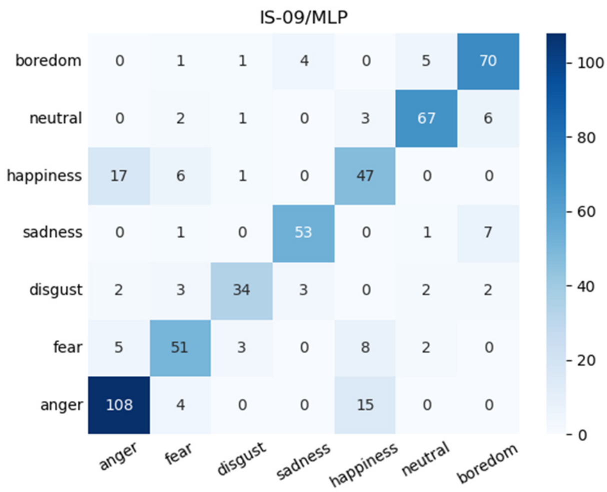
IS-09/MLP
boredom 0 1 1 4 0 5 70
neutral 0 2 1 0 3 67 6
happiness 17 6 1 0 47 0 0
sadness 0 1 0 53 0 1 7
disgust 2 3 34 3 0 2 2
fear 5 51 3 0 8 2 0
anger 108 4 0 0 15 0 0
anger fear disgust sadness happiness neutral boredom
100 80 60 40 20 0

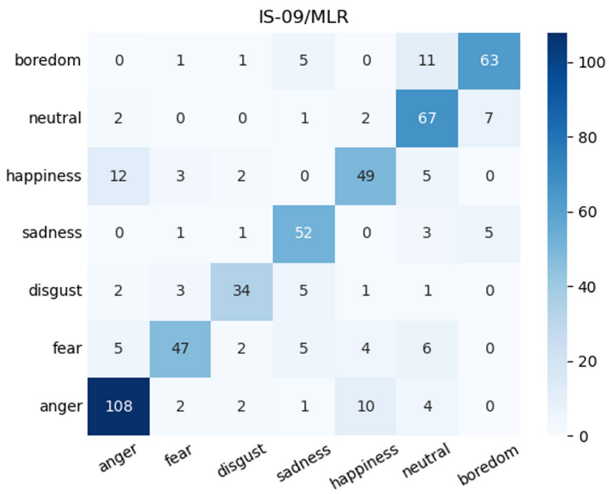
IS-09/MLR
boredom 0 1 1 5 0 11 63
neutral 2 0 0 1 2 67 7
happiness 12 3 2 0 49 5 0
sadness 0 1 1 52 0 3 5
disgust 2 3 34 5 1 1 0
fear 5 47 2 5 4 6 0
anger 108 2 2 1 10 4 0
anger fear disgust sadness happiness neutral boredom
100 80 60 40 20 0

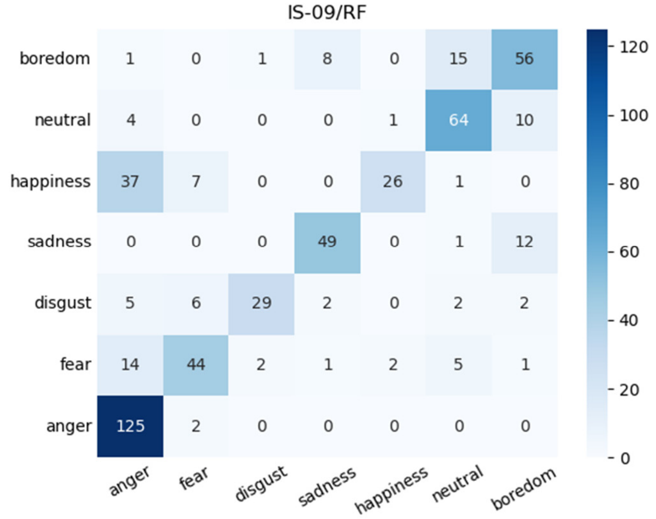
IS-09/RF
boredom 1 0 1 8 0 15 56
neutral 4 0 0 0 1 64 10
happiness 37 7 0 0 26 1 0
sadness 0 0 0 49 0 1 12
disgust 5 6 29 2 0 2 2
fear 14 44 2 1 2 5 1
anger 125 2 0 0 0 0 0
anger fear disgust sadness happiness neutral boredom
120 100 80 60 40 20 0

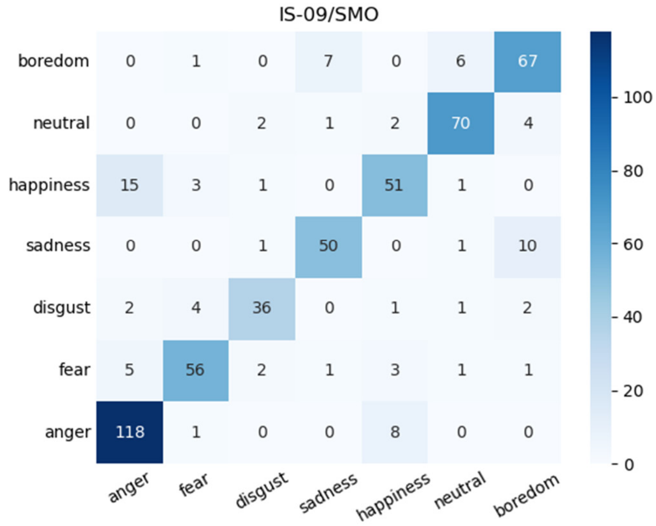
IS-09/SMO
boredom 0 1 0 7 0 6 67
neutral 0 0 2 1 2 70 4
happiness 15 3 1 0 51 1 0
sadness 0 0 1 50 0 1 10
disgust 2 4 36 0 1 1 2
fear 5 56 2 1 3 1 1
anger 118 1 0 0 8 0 0
anger fear disgust sadness happiness neutral boredom
100 80 60 40 20 0

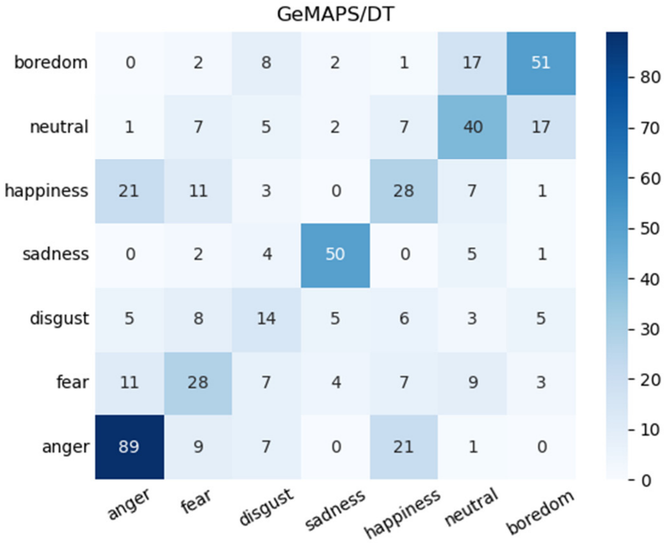
GeMAPS/DT
boredom 0 2 8 2 1 17 51
neutral 1 7 5 2 7 40 17
happiness 21 11 3 0 28 7 1
sadness 0 2 4 50 0 5 1
disgust 5 8 14 5 6 3 5
fear 11 28 7 4 7 9 3
anger 89 9 7 0 21 1 0
anger fear disgust sadness happiness neutral boredom
80 70 60 50 40 30 20 10 0

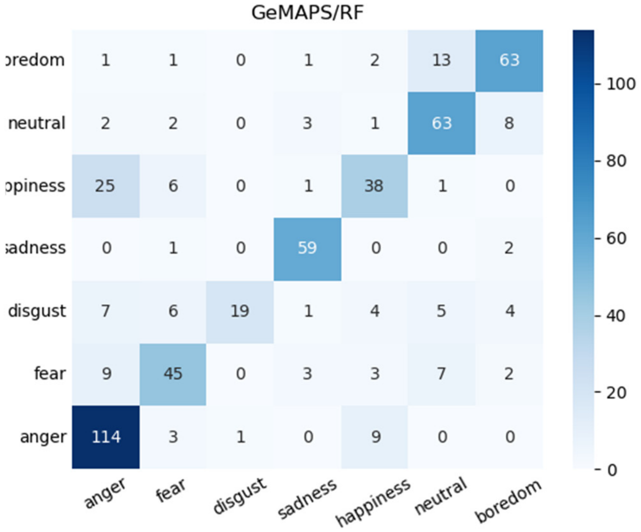
GeMAPS/RF
oredom 1 1 0 1 2 13 63
neutral 2 2 0 3 1 63 8
opiness 25 6 0 1 38 1 0
adness 0 1 0 59 0 0 2
disgust 7 6 19 1 4 5 4
fear 9 45 0 3 3 7 2
anger 114 3 1 0 9 0 0
anger fear disgust sadness happiness neutral boredom
100 80 60 40 20 0

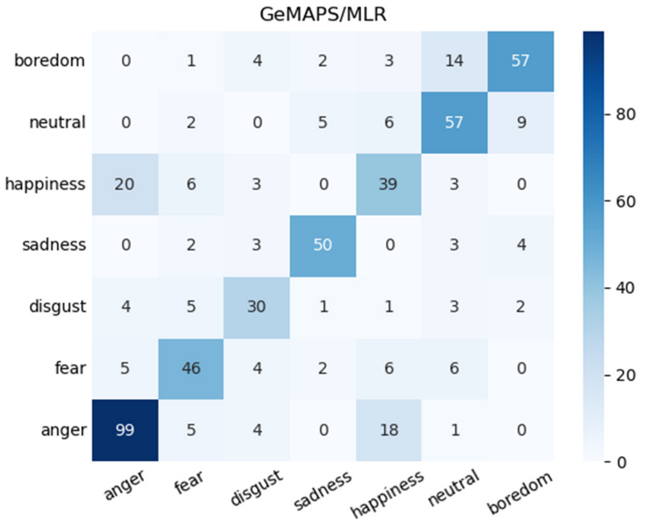
GeMAPS/MLR
boredom 0 1 4 2 3 14 57
neutral 0 2 0 5 6 57 9
happiness 20 6 3 0 39 3 0
sadness 0 2 3 50 0 3 4
disgust 4 5 30 1 1 3 2
fear 5 46 4 2 6 6 0
anger 99 5 4 0 18 1 0
anger fear disgust sadness happiness neutral boredom
80 60 40 20 0

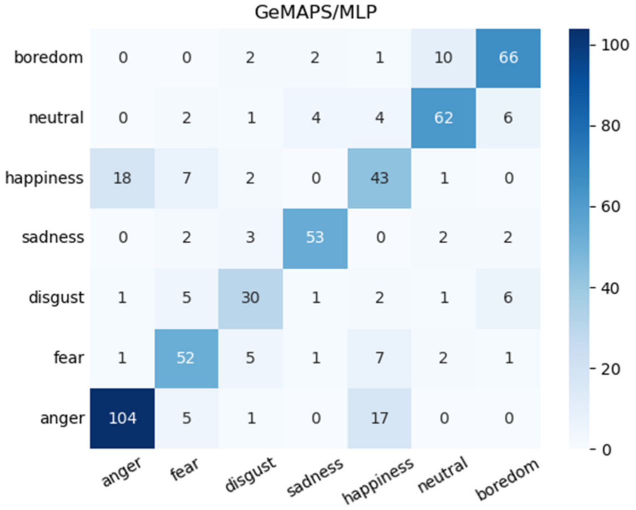
GeMAPS/MLP
boredom 0 0 2 2 1 10 66
neutral 0 2 1 4 4 62 6
happiness 18 7 2 0 43 1 0
sadness 0 2 3 53 0 2 2
disgust 1 5 30 1 2 1 6
fear 1 52 5 1 7 2 1
anger 104 5 1 0 17 0 0
anger fear disgust sadness happiness neutral boredom
100 80 60 40 20 0

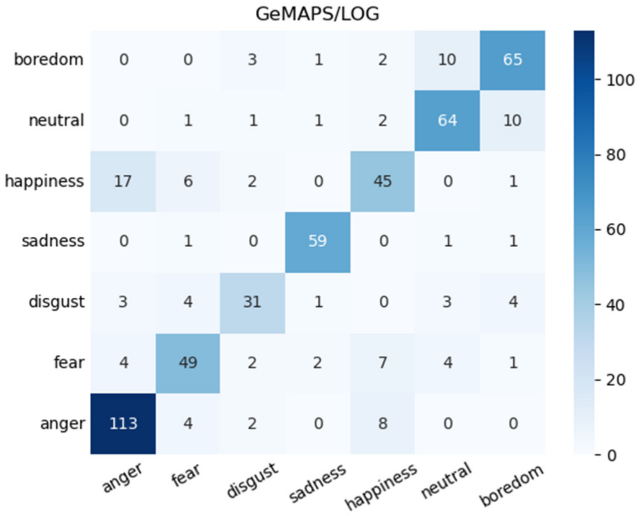
GeMAPS/LOG
boredom 0 0 3 1 2 10 65
neutral 0 1 1 1 2 64 10
happiness 17 6 2 0 45 0 1
sadness 0 1 0 59 0 1 1
disgust 3 4 31 1 0 3 4
fear 4 49 2 2 7 4 1
anger 113 4 2 0 8 0 0
anger fear disgust sadness happiness neutral boredom
100 80 60 40 20 0

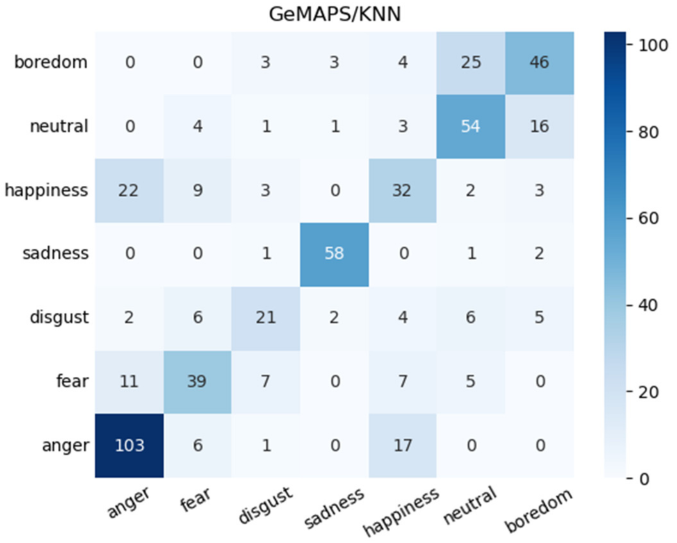
GeMAPS/KNN
boredom 0 0 3 3 4 25 46
neutral 0 4 1 1 3 54 16
happiness 22 9 3 0 32 2 3
sadness 0 0 1 58 0 1 2
disgust 2 6 21 2 4 6 5
fear 11 39 7 0 7 5 0
anger 103 6 1 0 17 0 0
anger fear disgust sadness happiness neutral boredom
100 80 60 40 20 0

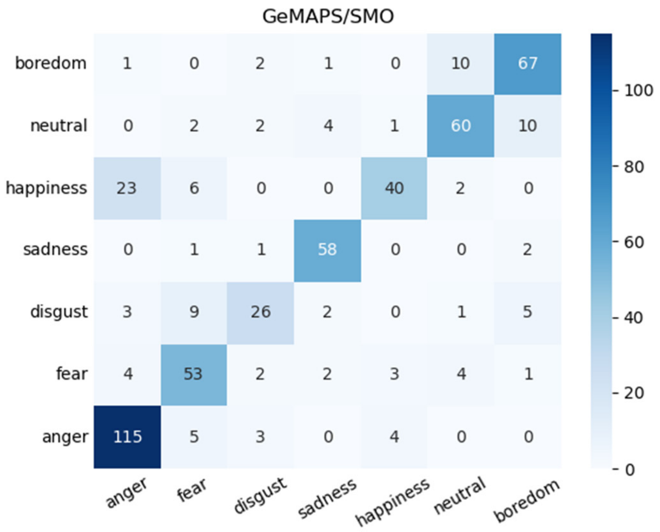
GeMAPS/SMO
boredom 1 0 2 1 0 10 67
neutral 0 2 2 4 1 60 10
happiness 23 6 0 0 40 2 0
sadness 0 1 1 58 0 0 2
disgust 3 9 26 2 0 1 5
fear 4 53 2 2 3 4 1
anger 115 5 3 0 4 0 0
anger fear disgust sadness happiness neutral boredom
100 80 60 40 20 0

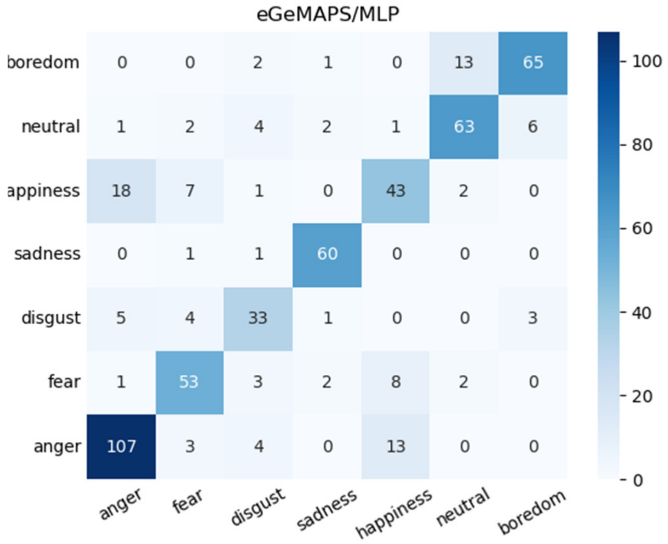
eGeMAPS/MLP
boredom 0 0 2 1 0 13 65
neutral 1 2 4 2 1 63 6
appiness 18 7 1 0 43 2 0
sadness 0 1 1 60 0 0 0
disgust 5 4 33 1 0 0 3
fear 1 53 3 2 8 2 0
anger 107 3 4 0 13 0 0
anger fear disgust sadness happiness neutral boredom
100 80 60 40 20 0

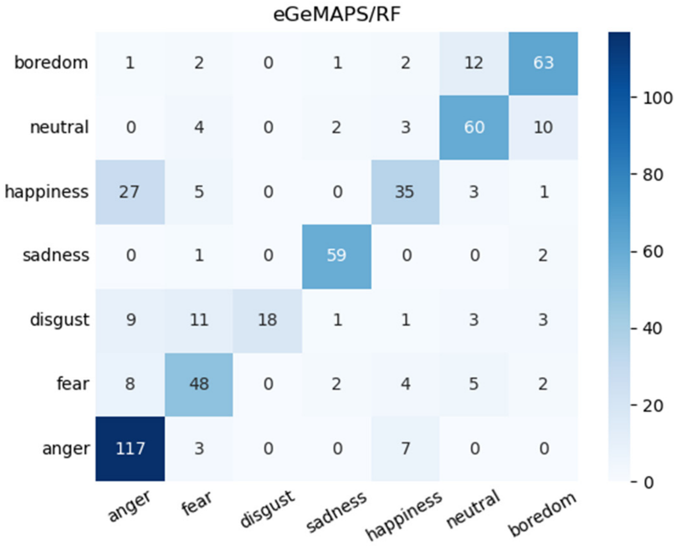
eGeMAPS/RF
boredom 1 2 0 1 2 12 63
neutral 0 4 0 2 3 60 10
happiness 27 5 0 0 35 3 1
sadness 0 1 0 59 0 0 2
disgust 9 11 18 1 1 3 3
fear 8 48 0 2 4 5 2
anger 117 3 0 0 7 0 0
anger fear disgust sadness happiness neutral boredom
100 80 60 40 20 0

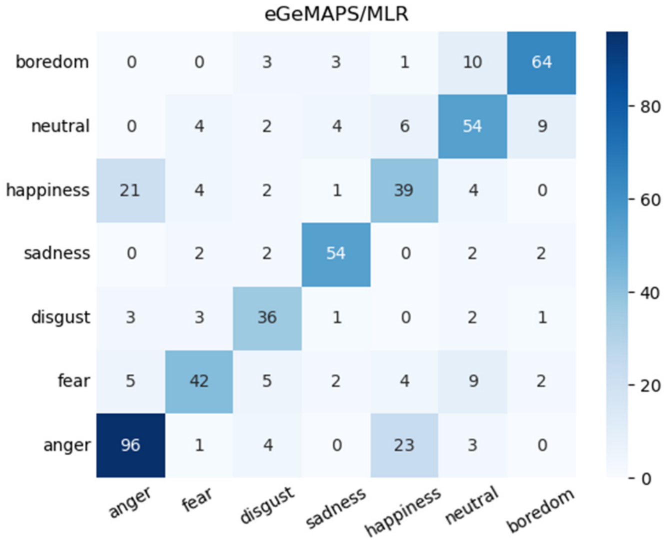
eGeMAPS/MLR
boredom 0 0 3 3 1 10 64
neutral 0 4 2 4 6 54 9
happiness 21 4 2 1 39 4 0
sadness 0 2 2 54 0 2 2
disgust 3 3 36 1 0 2 1
fear 5 42 5 2 4 9 2
anger 96 1 4 0 23 3 0
anger fear disgust sadness happiness neutral boredom
80 60 40 20 0

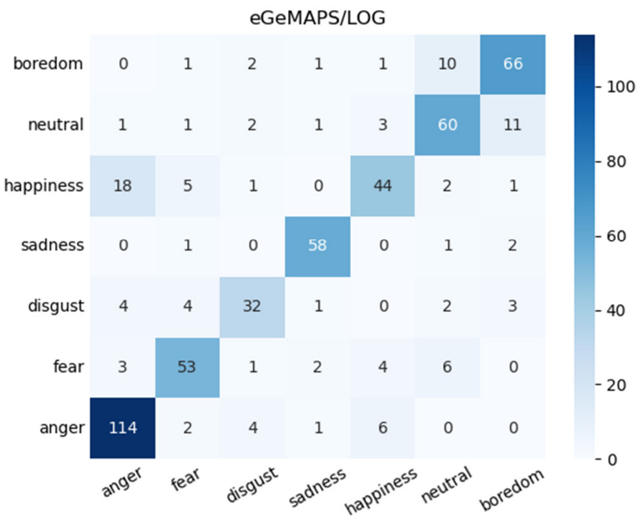
eGeMAPS/LOG
boredom 0 1 2 1 1 10 66
neutral 1 1 2 1 3 60 11
happiness 18 5 1 0 44 2 1
sadness 0 1 0 58 0 1 2
disgust 4 4 32 1 0 2 3
fear 3 53 1 2 4 6 0
anger 114 2 4 1 6 0 0
anger fear disgust sadness happiness neutral boredom
0 20 40 60 80 100

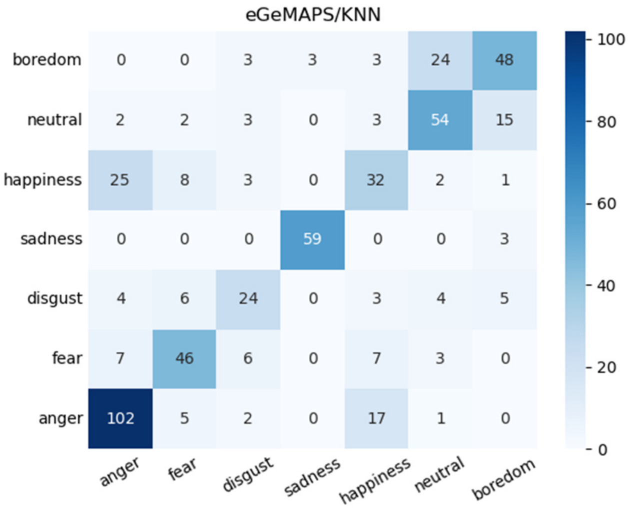
eGeMAPS/KNN
boredom 0 0 3 3 3 24 48
neutral 2 2 3 0 3 54 15
happiness 25 8 3 0 32 2 1
sadness 0 0 0 59 0 0 3
disgust 4 6 24 0 3 4 5
fear 7 46 6 0 7 3 0
anger 102 5 2 0 17 1 0
anger fear disgust sadness happiness neutral boredom
0 20 40 60 80 100

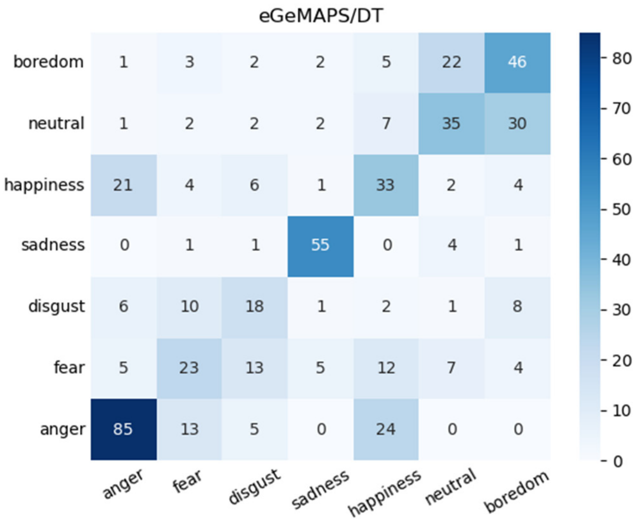
eGeMAPS/DT
boredom 1 3 2 2 5 22 46
neutral 1 2 2 2 7 35 30
happiness 21 4 6 1 33 2 4
sadness 0 1 1 55 0 4 1
disgust 6 10 18 1 2 1 8
fear 5 23 13 5 12 7 4
anger 85 13 5 0 24 0 0
anger fear disgust sadness happiness neutral boredom
0 10 20 30 40 50 60 70 80

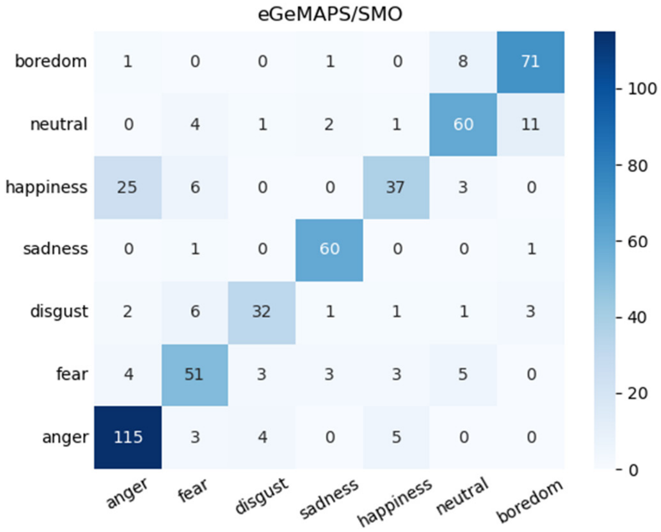
eGeMAPS/SMO
boredom 1 0 0 1 0 8 71
neutral 0 4 1 2 1 60 11
happiness 25 6 0 0 37 3 0
sadness 0 1 0 60 0 0 1
disgust 2 6 32 1 1 1 3
fear 4 51 3 3 3 5 0
anger 115 3 4 0 5 0 0
anger fear disgust sadness happiness neutral boredom
0 20 40 60 80 100
